# Age‐dependent heat shock hormesis to HSF‐1 deficiency suggests a compensatory mechanism mediated by the unfolded protein response and innate immunity in young *Caenorhabditis elegans*

**DOI:** 10.1111/acel.14246

**Published:** 2024-06-19

**Authors:** Dániel Kovács, János Barnabás Biró, Saqib Ahmed, Márton Kovács, Tímea Sigmond, Bernadette Hotzi, Máté Varga, Viktor Vázsony Vincze, Umar Mohammad, Tibor Vellai, János Barna

**Affiliations:** ^1^ Department of Genetics ELTE Eötvös Loránd University Budapest Hungary; ^2^ HUN‐REN‐ELTE Genetics Research Group Eötvös Loránd University Budapest Hungary

**Keywords:** autophagy, *C. Elegans*, cellular stress response, heat shock factor 1, heat shock proteins, heat shock response, hormesis, innate immunity, insulin‐like signaling pathway, intracellular pathogen response, proteostasis, *skn‐1*, thermotolerance, unfolded protein response

## Abstract

The transcription factor HSF‐1 (heat shock factor 1) acts as a master regulator of heat shock response in eukaryotic cells to maintain cellular proteostasis. The protein has a protective role in preventing cells from undergoing ageing, and neurodegeneration, and also mediates tumorigenesis. Thus, modulating HSF‐1 activity in humans has a promising therapeutic potential for treating these pathologies. Loss of HSF‐1 function is usually associated with impaired stress tolerance. Contrary to this conventional knowledge, we show here that inactivation of HSF‐1 in the nematode *Caenorhabditis elegans* results in increased thermotolerance at young adult stages, whereas HSF‐1 deficiency in animals passing early adult stages indeed leads to decreased thermotolerance, as compared to wild‐type. Furthermore, a gene expression analysis supports that in young adults, distinct cellular stress response and immunity‐related signaling pathways become induced upon HSF‐1 deficiency. We also demonstrate that increased tolerance to proteotoxic stress in HSF‐1‐depleted young worms requires the activity of the unfolded protein response of the endoplasmic reticulum and the SKN‐1/Nrf2‐mediated oxidative stress response pathway, as well as an innate immunity‐related pathway, suggesting a mutual compensatory interaction between HSF‐1 and these conserved stress response systems. A similar compensatory molecular network is likely to also operate in higher animal taxa, raising the possibility of an unexpected outcome when HSF‐1 activity is manipulated in humans.

## Graphical abstract

Inactivating HSF‐1 (heat shock factor 1), the master regulator of heat shock response, paradoxically increases the thermotolerance of the nematode C. elegans in an age‐dependent manner. In the absence of a functional HSF‐1 distinct cellular stress response and immunity‐related signaling pathways become induced. The activity of the unfolded protein response of the endoplasmic reticulum and the SKN‐1/Nrf2‐mediated oxidative stress response pathway, as well as an innate immunity‐related pathway, are required for the increase in thermotolerance.

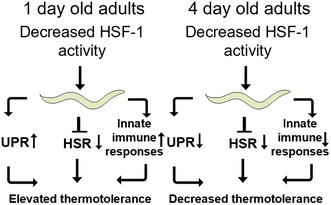

**Abbreviations**
*C. elegans*
: *Caenorhabditis elegans*
DEGs: differentially regulated genes
*E. coli*
: *Escherichia coli*
ER: endoplasmic reticulum
EV: empty vector
FUdR: 5‐fluoro‐2′‐deoxyuridine
GFP: green fluorescent protein
GSEA: Gene Set Enrichment Analysis
HSF1: heat shock factor 1
HSF‐1ΔTAD: C‐terminally truncated HSF1
HSP: heat shock protein
HSR: heat shock response
IlS: insulin‐like signaling
IPR: intracellular pathogen response
*N. paresis*
: *Nematocida parisii*
qPCR: quantitative polymerase chain reaction
RNAi: RNA interference
RNAseq: RNA sequencing
UPR^ER^
: unfolded protein response of the endoplasmic reticulum

## 1 INTRODUCTION

Proteins of living organisms are optimized to function within a narrow temperature range; therefore, a slight increase in temperature can lead to the disruption of protein homeostasis (proteostasis). Upon heat stress, conserved cell protective mechanisms are induced to preserve proteostasis (Kourtis & Tavernarakis, 2011; Higuchi‐Sanabria, Frankino, et al., 2018). These mechanisms include the unfolded protein response of the endoplasmic reticulum (UPR^ER^), the insulin/IGF‐1 (insulin‐like growth factor 1) signaling (IlS) pathway, autophagy (cellular self‐eating), and the heat shock response (HSR). The HSR leads to a robust activation of genes encoding heat shock proteins (HSPs). HSPs function as molecular chaperones that help refold or degrade damaged proteins, thereby contributing to the protection of cells from protein‐damaging stress (Hipp et al., 2019; Somogyvári et al., 2022).

The evolutionarily conserved heat shock transcription factor HSF‐1 acts as a master regulator of HSR (Joutsen & Sistonen, 2019; Kovács et al., 2022). Upon proteotoxic stress, HSF‐1 becomes activated via trimerization and phosphorylation, then translocates into the nucleus to promote the transcription of genes encoding HSPs (Akerfelt et al., 2010). Hence, HSF‐1 has a crucial role in maintaining proteostasis, specifically in the cytoplasm during normal development as well as under conditions of stress and aging (Barna et al., 2012, 2018; Hsu et al., 2003; Kovács et al., 2022; Li et al., 2017; Morimoto, 2020). The cell protective role of HSF‐1 was also described in malignant tumors, designating the protein as a promising target for cancer therapy (Dai & Sampson, 2016; Dong et al., 2019; Whitesell & Lindquist, 2009). The therapeutic potential of HSF‐1 is also significant in preventing ageing and in age‐related neurodegenerative diseases (Roos‐Mattjus & Sistonen, 2021). This raises the importance of determining the effects of HSF‐1 depletion at the organismal level.

**FIGURE 1:**
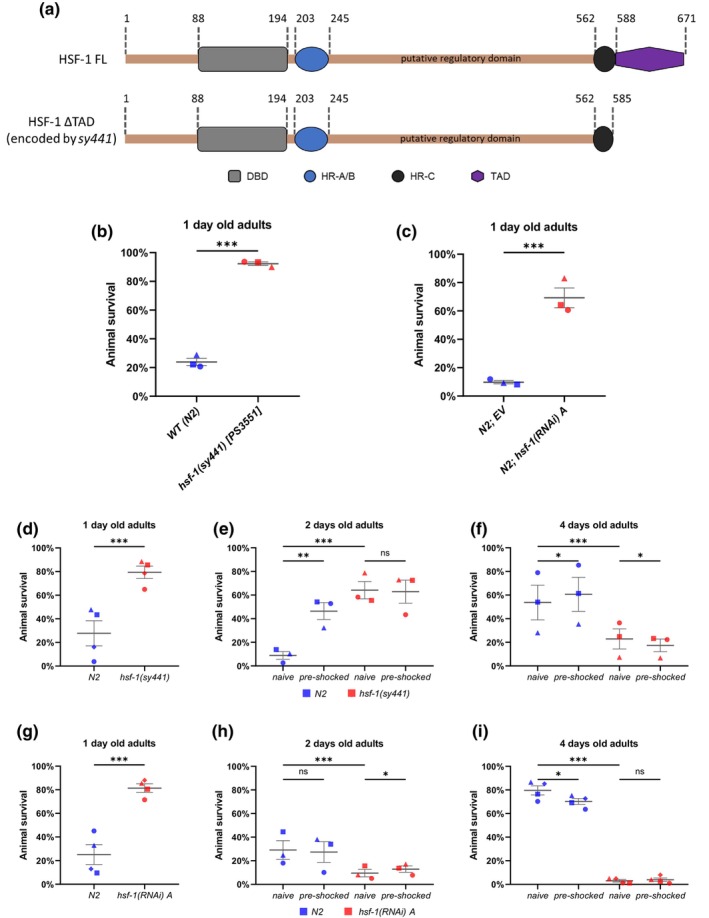
Inactivation of HSF‐1 results in increased thermotolerance in young adult *C. elegans*. (a) Domain organization of full length (FL) and C‐terminally truncated (ΔTAD) CeHSF‐1 proteins. DBD: DNA binding domain, binds the consensus HSE sequence; HR‐A/B oligomerization domain: heptad repeat A (HR‐A) and heptad repeat B (HR‐B) leucine‐zipper domains mediate the trimerization of HSF1. HR‐C heptad repeat domain keeps HSF1 in a monomeric form, due to its interaction with the HR‐A/B domain. TAD: transactivation domain, responsible for transcriptional activation. (b) *hsf‐1*(*sy441*) [PS3551] mutant animals have an increased thermotolerance compared to wild‐type at the 1 day old adult stage (animals were shifted from 20°C to 35°C for 5 h). (c) The increase in thermotolerance can be observed in animals treated with a type of *hsf‐1* RNAi and shifted from 20°C to 35°C for 5 h. (EV = empty vector, control for the RNAi treatment) (d) At the 1st day of adulthood *hsf‐1*(*sy441*) mutant animals tolerate heat stress better than wild‐type (animals were shifted from 20°C to 35°C for 5 h). (e) 2 days old adult *hsf‐1*(*sy441*) mutants still had an increased thermotolerance compared to wild‐type. Preconditioning (heat shock for 30 min. at 35°C, 18 h before the thermotolerance assay) increased the thermotolerance of wild‐type but had no effect on the *hsf‐1*(*sy441*) mutants. (f) By the 4th day of adulthood, the increased thermotolerance of *hsf‐1*(*sy441*) animals disappeared. At this stage the pre‐shock did not increase the survival of either wild‐type or *hsf‐1*(*sy441*) mutant animals. (g) Downregulating *hsf‐1* by using RNA interference also increased the survival rate of 1‐day old *C. elegans* adults under heat‐shock conditions (animals were shifted from 20°C to 35°C for 6 h). (h) 2 day old adult animals treated with *hsf‐1* RNAi had lower thermotolerance compared to control. In this case preconditioning has no effect on either pre‐shocked or naive animals. (i) At the 4 day old adult stage the control group is clearly more tolerant to heat stress than *hsf‐1*(*RNAi*) animals. Pre‐shock has no effect on the animals treated with *hsf‐1* dsRNA, while it slightly lowers the survival rate of untreated *C. elegans*. (b–i) Thermotolerance following a 5 h‐long (6 h long in case of RNAi treatment) heat shock at 35°C. *n* > 3 replicates of 50 animals per strain. Individual data points represent independent trials (the different biological replicates are indicated by different shapes), lines represent means. Significance compared to wild type control was determined using Cochran–Mantel–Haenszel test; * = *p* < 0.05 ** = *p* < 0.01 *** = *p* < 0.001; error bars represent ± SEM. Source data underlying Figure 1b–i are provided in Table S1. EV = empty vector; *hsf‐1* = heat shock factor 1; RNAi = RNA interference; SEM = standard error of the mean.

Despite extensive research on HSF‐1, there is a discrepancy regarding the effect of HSF‐1 deficiency on the thermotolerance of *C. elegans* (Kyriakou et al., 2022). To date, only two mutant alleles of *hsf‐1* are available. *ok600* is a deletion (possibly null) allele, and animals homozygous for this allele arrest development at the L2‐L3 larval stages (Morton & Lamitina, 2013). *sy441* is a reduction‐of‐function allele predicted to encode an HSF‐1 protein that lacks the transactivation domain, thereby being considered to be defective in heat shock‐induced transcriptional activation (Figure 1a) (Hajdu‐Cronin et al., 2004). Several groups have shown that *hsf‐1*(*sy441*) hypomorph mutants and *hsf‐1*(*RNAi*) (RNA interference) animals are more sensitive to heat shock than wild‐type (Finger et al., 2021; Prahlad et al., 2008; Steinkraus et al., 2008). Controversially, it has also been reported that there is no significant difference between the thermotolerance of wild‐type and *hsf‐1*(*sy441*) mutant animals (Kourtis et al., 2012; McColl et al., 2010). Intriguingly, in some cases, *hsf‐1*(*sy441*) mutant and *hsf‐1*(*RNAi*) animals were shown to have increased thermotolerance as compared to wild‐type animals (Golden et al., 2020; Morton, 2013).

In this work, we clarified this discrepancy by showing that the thermotolerance of animals defective for HSF‐1 activity changes at the early adult ages. 1‐day‐old adult worms displayed increased tolerance to heat stress, whereas the thermotolerance of adults at later stages was reduced, as compared to the wild‐type. Furthermore, in 1‐day‐old adults deficient in HSF‐1 function, the activity of genes involved in the innate immune system and UPR^ER^ was elevated relative to control, while induction of these genes was abolished in older animals. We also show that the UPR^ER^ and innate immunity‐related signaling pathway are required for enhanced heat stress resistance in young HSF‐1‐deficient animals. Finally, a C‐terminally truncated form of HSF‐1, encoded by *hsf‐1*(*sy441*), retains its activity to upregulate heat shock protein‐encoding genes, preferentially in the intestine.

## 2 RESULTS

### 2.1 HSF‐1 deficiency results in increased thermotolerance at the first day of adulthood

We determined thermotolerance in *hsf‐1*(*sy441*) mutant and *hsf‐1*(*RNAi*) animals at the stage of 1‐day‐old adulthood, using a modified protocol based on the work of Zevian and Yanovitz Zevian and Yanowitz (2014). Surprisingly, we found that both *hsf‐1*(*sy441*) mutant animals and *hsf‐1*(*RNAi*) nematodes tolerate heat stress significantly better than control (wild‐type worms and animals fed with bacteria expressing the empty RNAi vector only, respectively) (Figure 1b,c). To exclude the possibility that the increased thermotolerance observed was caused by a background mutation or the off‐target effect of HSF‐1 depletion, we repeated this set of experiments using a strain that was outcrossed six times with the wild‐type [TTV450 {*hsf‐1*(*sy441*)*x6*}] and another RNAi construct against *hsf‐1* and obtained similar results (Figure S1a,b). We also observed that worms grown on HT115 bacteria (this bacterial strain is commonly used in feeding RNAi experiments) have a higher thermotolerance, a phenomenon that has been described previously (Revtovich et al., 2019). Therefore, we applied a 6‐h‐longer heat shock protocol in all survival assays coupled withRNAi treatment, since after 5 h at 35°C, the survival of control and hsf‐1(RNAi) worms was almost 100% and no significant difference in survival was found between the two groups (Figure S1c). However, when animals were heat‐shocked for 6 h, there was a significant difference in thermotolerance between the survival of control and hsf‐1(RNAi) nematodes. To exclude the possibility that these results were due to some peculiarity of the protocol, we applied another thermotolerance protocol, which monitors the survival of animals exposed to stress at every hour (Bar‐Ziv et al., 2020). Nevertheless, at the first day of adulthood, HSF‐1‐depleted nematodes proved to be more resistant to heat stress than the control at the same stage (Figure S1d,e). It was shown recently that there is a significant decrease in stress tolerance at an early adult stage, the onset of egg‐ (embryo) laying (Labbadia & Morimoto, 2015). This raises the possibility that the increased stress tolerance of worms defective for HSF‐1 function is a consequence of a slower developmental rate as compared to wild‐type. At the time when the thermotolerance assay was performed, wild‐type nematodes had already passed this stage while animals deficient in HSF‐1 function were still at the stress resistant developmental stage. To test this possibility, we determined the thermotolerance of *hsf‐1*(*sy441*) mutant and *hsf‐1*(*RNAi*) adults at the time of 62, 64, and 71 h after the eggs were laid. According to our results the thermotolerance of *hsf‐1*(*sy441*) mutant and *hsf‐1*(*RNAi*) worms was elevated at each time point compared to the corresponding controls (Figure S1f,g). We conclude that 1‐day‐old nematodes with HSF‐1 deficiency exhibit an elevated resistance to heat stress as compared with control.

### 2.2 The effect of HSF‐1 deficiency on thermotolerance depends on age

It has been demonstrated that 2‐day‐old *hsf‐1*(*sy441*) mutant adult animals are as tolerant to heat stress as wild‐type at the same stage (McColl et al., 2010). Other groups have shown that *hsf‐1*(*sy441*) mutant nematodes at mid adult stages are more sensitive to heat stress than wild‐type animals (Finger et al., 2021; Prahlad et al., 2008; Steinkraus et al., 2008). These data suggest that the effect of HSF‐1 deficiency on the thermotolerance of nematodes changes at the early adult stages. To test this possibility, we determined the thermotolerance of 1‐, 2‐ and 4‐day‐old adult worms. Due to the severe egg‐laying defective (Egl) phenotype of HSF‐1‐deficient animals, we sterilized worms by growing them on plates supplemented with 5‐fluoro‐2′‐deoxyuridine (FUdR), a potent inhibitor of cell proliferation (Sutphin & Kaeberlein, 2009). Application of the compound repressed premature death in Egl adult hermaphrodites, which was due to young larvae hatched inside their uterus. Figure 1 shows that at the first day of adulthood, the survival of both *hsf‐1*(*sy441*) mutant and *hsf‐1*(*RNAi*) animals exposed to heat stress is significantly higher than the corresponding controls (Figure 1d,g). At the second day of adulthood, *hsf‐1*(*sy441*) mutants still tolerated heat stress better than wild‐type nematodes, but the heat tolerance of *hsf‐1*(*RNAi*) animals was lower than that of control fed with bacteria expressing the empty RNAi vector only (Figure 1e,h). This discrepancy was also reflected by the hormetic effect of pre‐stressed worms raised on *E. coli* OP50 versus HT115 (RNAi feeding) bacteria; while a mild stress (35°C, 30 min) 18 h before the assay enhanced the stress tolerance of 2‐day‐old adults fed on OP50 bacteria, the same preconditioning had no effect on the heat tolerance of worms grown on HT115 bacteria (Figure 1e,h). Impact of diet on the survival of *C. elegans* after exposure to heat stress has also been described earlier (Revtovich et al., 2019).

In the case of 4‐day‐old adults, the wild‐type strain was more tolerant to heat stress than *hsf‐1*(*sy441*) mutants (Figure 1f). We observed the same effect when repeating the experiment with *hsf‐1*(*RNAi*) animals at the same adult stage (Figure 1i). As a control test, we investigated whether FUdR influences these results. To this end, we determined the thermotolerance of control and *hsf‐1*(*RNAi*) animals grown on agar plates supplemented with no FUdR by carefully selecting animals that did not show the Egl phenotype. We obtained results that were highly similar to those observed under conditions of applying FUdR. Therefore, we concluded that FUdR does not interfere with the effect of HSF‐1 deficiency on the thermotolerance of animals at the stages assayed (Figure S2b). Taken together, these results indicate that HSF‐1 deficiency inversly influences thermotolerance at early (1 day) and mid (2–4 days) adult stages.

### 2.3 Genes involved in stress response‐ and innate immunity‐related pathways are upregulated in *hsf‐1*(*sy441*) mutant animals

**FIGURE 2:**
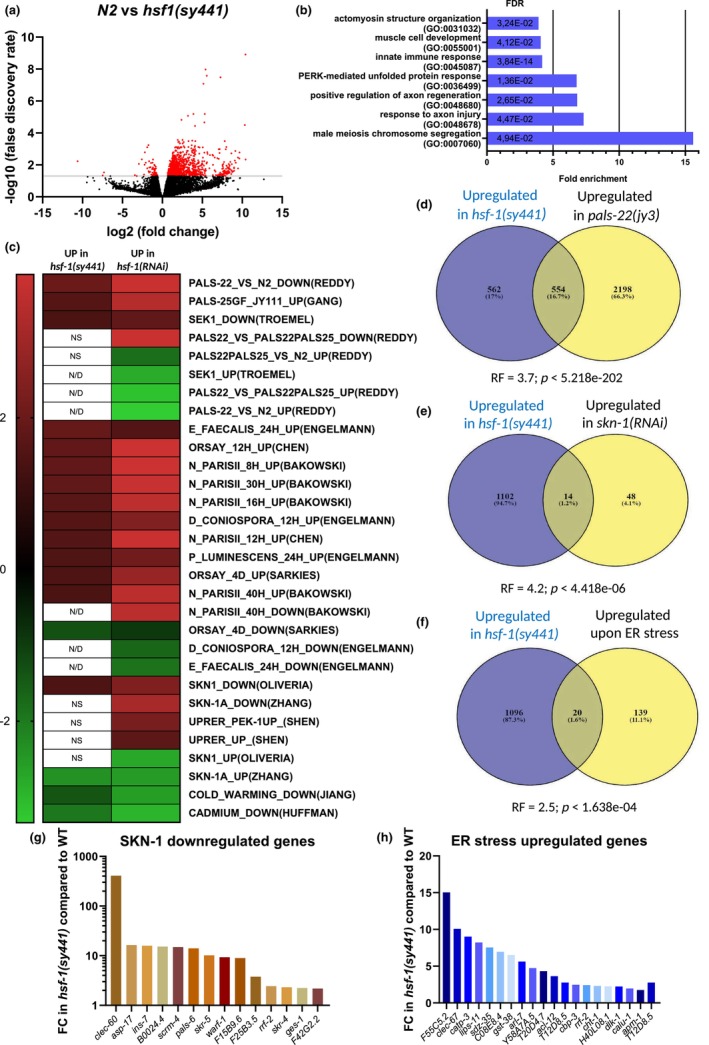
Genes involved in distinct stress and immunity related pathways are upregulated in *hsf‐1*(*sy441*) mutant animals. (a) RNA‐Seq volcano plot showing log_2_‐fold change in expression levels of genes (FDR <0.05) differentially regulated (1116 up‐regulated; 72 down‐regulated) in 1 day old *hsf‐1*(*sy441*) mutant adults compared to wild‐type (N2) at 20°C. (b) GO enrichment analysis shows that genes involved in PERK‐mediated UPR and related to innate immune response are enriched among differentially expressed genes. Horizontal axis shows the fold enrichment. (c) Genes transcriptionally upregulated in *hsf‐1*(*sy441*) mutant and *hsf‐1*(*RNAi*) genetic backgrounds correlate with genes that are up‐ or downregulated in response to pathogens, abiotic stresses or conserved regulators of cellular stress response pathways. Analysis was performed using the GSEA 4.3.2 software package (see the Materials and Methods). Correlation was quantified as a Normalized Enrichment Score (NES). NES is positive (red) when a gene set show correlation with upregulated genes, while NES is negative (green) when a gene set show correlation with downregulated genes. Cells are white if no significant correlation was detected (FDR >0.25, or nominal *p*‐value >0.05) or NES values are not available (N/D). Gene sets used are available in Table S5. For more details, see Table S6. (d–f) Venn diagrams showing that genes upregulated in *hsf‐1*(*sy441*) mutants compared to wild‐type animals have a significant overlap with genes upregulated in *pals‐22*(*jy3*) mutants compared to wild‐type (RF = 3.7; *p* < 5.218e‐202) (d), with genes upregulated in *skn‐1*(*RNAi*) compared to EV control (RF = 4.2; *p* < 4.418e‐06) (E) or with genes induced upon ER stress that are dependent on IRE‐1, PEK‐1 or ATF‐6 signaling (RF = 2.5; *p* < 1.638e‐04) (f). (g) Diagram showing the relative levels of genes significantly upregulated in both, *hsf‐1*(*sy441*) mutant and *skn‐1*(*RNAi*) backgrounds. FC and FDR were calculated by edgeR (see Materials and Methods and Table S4). (h) Diagram showing the relative levels of genes significantly upregulated in both, *hsf‐1*(*sy441*) mutant and upon ER stress. FC and FDR was calculated by edgeR (see Materials and Methods and Table S5). Source data underlying Figure 2a,b are provided in Table S4. Source data underlying Figure 2c are provided in Table S7. Source data underlying Figure 2d–f are provided in Tables S5 and S8; FDR = false discovery rate; *hsf‐1* = heat shock factor 1; RNAi = RNA interference; GO = Gene Ontology.

To explore how HSF‐1 deficiency can lead to increased thermotolerance at the first day of adulthood, we performed an RNAseq analysis in wild type versus *hsf‐1*(*sy441*) mutant genetic backgrounds under normal (20°C) and heat‐shocked conditions. We found that under physiological conditions the expression of several genes becomes elevated in *hsf‐1*(*sy441*) mutants as compared to wild type (Figure 2a, Tables S4 and S5).

An overrepresentation test using Panther (Mi et al., 2021) showed that genes involved in male meiosis chromosome segregation, the regulation of axon regeneration, PERK‐mediated unfolded protein response, innate immunity, muscle cell development, and actomyosin structure organization are overrepresented among those activated in the *hsf‐1*(*sy441*) mutant genetic background (Figure 2b). Interestingly, several stress‐response pathways were also upregulated in *hsf‐1*(*sy441*) mutant animals. These include, for example, the UPR^ER^ (e.g., *calu‐1*, *lips‐11*, and *warf‐1*), autophagy (e.g., *sqst‐1/p62*), ubiquitin‐dependent proteolysis (*skr‐4* and *5*) and IIS (e.g., *sod‐3* and *gst‐10*) (Table S5). Another group of differentially regulated genes is related to immune response, including lysozymes (*lys‐1*, *lys‐2*, *lys‐7*, and *lys‐8*), *cnc‐2* encoding for a bacteriocin, components of activator protein 1 (AP1), such as *jun‐1* and *fos‐1*, infection response *irg‐2*, fungus‐induced *fipr‐22 and fipr‐23*, CUB domain‐containing protein‐encoding *dod‐22* and *dct‐17*, and F‐box domain‐containing protein‐encoding *fbxa‐58* and *fbxa‐59* (Table S5).

Next, we compared differentially regulated genes (DEGs) in both *hsf‐1*(*sy441*) mutant and *hsf‐1*(*RNAi*) animals (Brunquell et al., 2016) to a gene set collection containing DEGs from published RNAseq data, using Gene Set Enrichment Analysis (GSEA) (Subramanian et al., 2005). These sets involve genes regulated in response to infection by a variety of pathogens, abiotic stresses, and conserved regulators of cellular stress response pathways (Table S5). The analysis revealed that in both *hsf‐1*(*sy441*) mutant and *hsf‐1*(*RNAi*) animals those genes are activated, which are either turned on during infection by various pathogens (e.g., Orsay virus, *N. parisii*), regulated by an innate immunity‐related proteins (e.g., SEK‐1, PALS‐22 and PALS‐25), or inhibited by Skn1/Nrf2 signaling (SKN‐1 mediates the response of nematodes to oxidative stress) (Figure 2c and Tables S6 and S7). We also found that genes upregulated in *hsf‐1*(*RNAi*) animals are enriched in the gene set upregulated by the ER stress inducer tunicamycin (Shen et al., 2005). This suggests that genes involved in the UPR^ER^ are induced in HSF‐1‐depleted animals (Figure 2c and Table S7).

Using available RNAseq data from the literature (see Table S8), we performed a Venn analysis and found that there is a significant overlap between genes upregulated in *hsf‐1*(*sy441*) and *pals‐22*(*jy3*) mutants (Figure 2d and Table S8). Similarly, several genes were commonly activated in the *hsf‐1*(*RNAi*) and *pals‐22*(*jy3*) mutant backgrounds (Table S8). PALS‐22 is a negative regulator of the intracellular pathogen response (IPR), a recently identified transcriptional response to several natural intracellular pathogens of *C. elegans* (Reddy et al., 2017, 2019). This finding implies that HSF‐1, either directly or indirectly, inhibits a significant number of genes involved in the IPR. Comparing gene sets downregulated by HSF‐1 and SKN‐1 led to the identification of genetic factors that are activated in both *hsf‐1*(*sy441*) mutant and *skn‐1*(*RNAi*) genetic backgrounds (Figure 2e,g and Table S8). Of note, genes involved in the UPR^ER^ and genes upregulated in the *hsf‐1*(*sy441*) mutant background exhibit a significant overlap (Figure 2f,h and Table S8). Together, HSF‐1 and SKN‐1 inhibit an overlapping set of genes, and several UPR‐related genes are activated in animals defective for HSF‐1 function.

### 2.4 Genes encoding for HSPs are upregulated in *hsf‐1*(*sy441*) mutant animals

**FIGURE 3:**
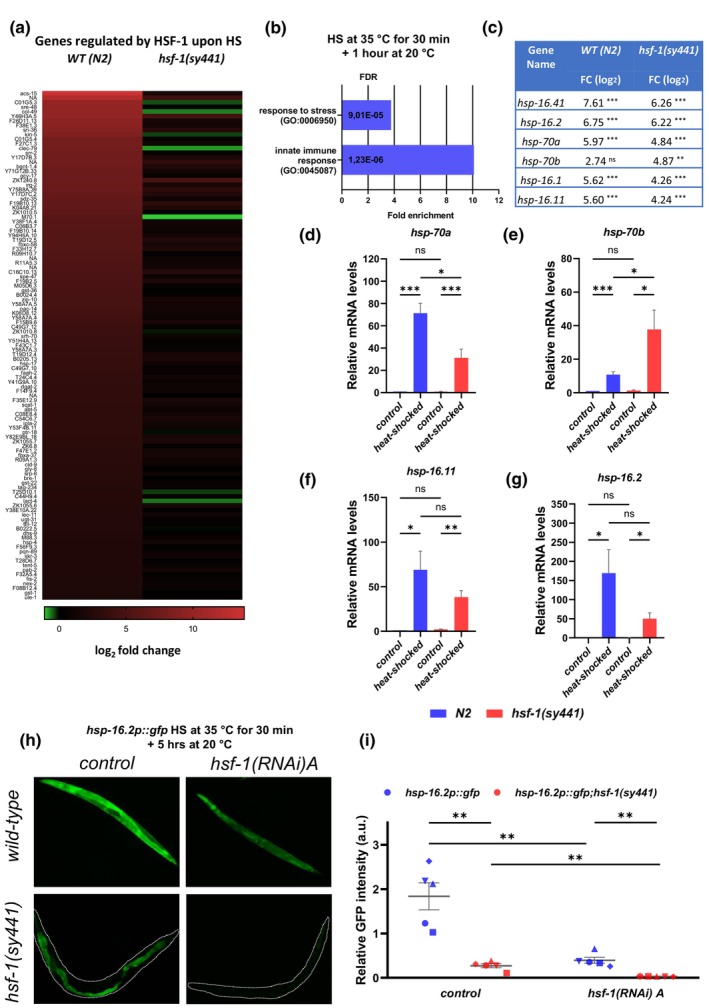
Heat shock genes are upregulated in *hsf‐1*(*sy441*) young adults upon robust heat shock. (a) Heat map showing the relative gene expression levels of differently regulated genes in wild type (*N2*) and *hsf‐1*(*sy441*) mutant 1‐day‐old adult nematodes following heat shock at 35°C for 30 min compared to the untreated control (b) GO enrichment analysis shows that upon heat‐shock genes related to stress responses are up‐regulated in 1 day old *hsf‐1*(*sy441*) mutant adults. Horizontal axis shows the fold enrichment. (c) Table showing heat shock protein coding genes upregulated in both, wild‐type, and *hsf‐1*(*sy441*) mutant animals. (d–g) Results of qPCR experiment showing that mRNA levels of heat shock protein coding genes *hsp‐70a*, *hsp‐70b*, *hsp‐16.2* and *hsp‐16.11* are induced in *hsf‐1*(*sy441*) mutants upon heat shock. The diagrams show the result of four replicates. *p* values were determined using Welch's *t*‐test * = *p* < 0.05 ** = *p* < 0.01 *** = *p* < 0.001; error bars represent + SEM (h) Representative fluorescent images showing expression of *hsp‐16.2p::GFP* reporter quantified in (i). Note that *hsp‐16.2p::GFP* is expressed exclusively in the intestinal cells in *hsf‐1*(*sy441*) mutants upon heat shock. (i) Plot showing that induction of *hsp‐16.2p::GFP* upon heat shock in *hsf‐1*(*sy441*) requires HSF‐1 activity. Three replicates of at least 55 animals per strain / trial were analyzed. Date points represent mean of independent trials, lines represent mean of means, *p* values were determined using Welch's *t*‐test * = *p* < 0.05 ** = *p* < 0.01 *** = *p* < 0.001; error bars represent ± SEM. Source data underlying Figure 4a–c are provided in Table S8. Source data underlying Figure 3d–i are provided in Table S9. EV = empty vector; FDR = false discovery rate; *hsf‐1* = heat shock factor 1; RNAi = RNA interference; GO = Gene Ontology; SEM = standard error of the mean.

Upon heat shock, genes involved in stress response and innate immune response are upregulated by HSF‐1 (Figure 3a,b). Interestingly, some of the genes coding for heat shock proteins were also upregulated in response to heat stress in the *hsf‐1*(*sy441*) mutant background (Figure 3a,c and Table S9). These genes include *hsp‐16*.*2*, *hsp‐70A*, and *hsp‐16.11*. A similar induction of *hsp* genes was previously observed in a *hsf‐1*(*sy441*) mutant background overexpressing HSF‐1ΔTAD transgene (Figure 3c) (Baird et al., 2014). The HSF‐1 allele *sy441* is considered to lack the transcription activation domain of the protein (Figure 1a) (Hajdu‐Cronin et al., 2004). This prompted us to confirm the results above by performing a qPCR (quantitative polymerase chain reaction) analysis using independently isolated mRNA samples from untreated control and heat‐shocked, wild‐type versus *hsf‐1*(*sy441*) mutant adults at the stage of 1 day of adulthood (Figure 3d–g). We also tested the expression of an *hsp‐16.2p::gfp* reporter in wild‐type versus *hsf‐1*(*sy441*) mutant animals exposed to heat shock. The results showed that expression of the reporter is decreased in *hsf‐1*(*sy441*) mutants as compared to wild‐type, but clearly elevated 5 h after the heat shock (Figure 3h,i). The induction was however suppressed when *hsf‐1*(*sy441*) mutant animals were treated with double‐stranded RNA specific to *hsf‐1* (Figure 3h,i), suggesting that heat shock‐induced *hsp‐16.2p::gfp* upregulation in the *hsf‐1*(*sy441*) mutant background is fully dependent on the activity of the C‐terminally truncated HSF‐1ΔTAD and not mediated by an alternative, heat stress‐sensitive pathway. Interestingly, the expression of the reporter was induced in the intestinal cells only (Figure 3h). These results indicate that truncated HSF‐1, lacking the transactivation domain, is still capable of activating the transcription of heat shock genes under conditions of robust heat shock, at least in the intestine.

### 2.5 Induction of stress response‐ and innate immunity‐related genes in *hsf‐1*(*sy441*) mutant nematodes depends on the stage of adulthood

**FIGURE 4:**
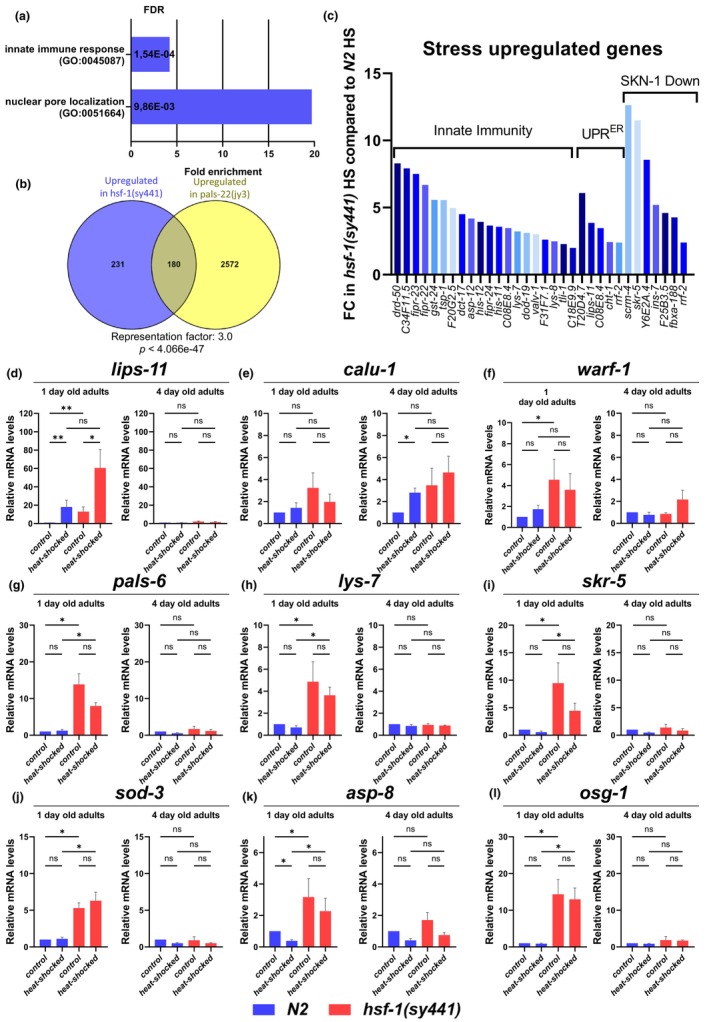
Induction of stress and immunity related genes in *hsf‐1*(*sy441*) mutant nematodes is age dependent. (a) Expression of several genes that are involved in innate immunity is overactivated in *hsf‐1*(*sy441*) mutants compared to the wild type following heat shock. (b) A set of these genes is overlaps with genes induced in *pals‐22*(*jy3*) mutants a negative regulator of intracellular pathogen response suggesting that HSF‐1 inhibits IPR related gene expression. (c) Genes that are upregulated in *hsf‐1*(*sy441*) mutants following heat shock include genes involved in innate immunity, UPR^ER^ and genes that are downregulated in a *skn‐1*(*−*) mutant background. (d–f) mRNA levels of the UPRER related genes *lips‐11*, *calu‐1* and *warf‐1* are higher in *hsf‐1*(*sy441*) mutants compared to the wild type. (g–h) The expression levels of genes involved in immunity (*pals‐6* and *lys‐7*) are also increased in 1‐day old *hsf‐1*(*sy441*) mutants, but this difference is diminished by 4th day of adulthood. (i–l) Genes related to various stress responses such as proteasomal degradation (*skr‐5*), the IlS pathway *sod‐3*, *asp‐8*, and oxidative stress (*osg‐1*) showed a similar expression pattern during ageing in *hsf‐1*(*sy441*) mutants compared to the wild type. (d–l) All these data indicate that the induction of stress and immunity related genes in *hsf‐1*(*sy441*) mutant nematodes is age‐dependent. Bars represent mean of at least three trials. Significance was determined using Mann–Whitney test; * = *p* < 0.05 ** = *p* < 0.01 *** = *p* < 0.001; error bars represent + SEM. Source data underlying Figure 2g–o are provided in Table S10.

We found that several stress response‐ and innate immunity‐related genes become induced in *hsf‐1*(*sy441*) mutants under physiological conditions (Figure 2b and Table S5) as well as following heat shock (Figure 4a and Table S9). A set of these genes overlaps with genes induced in *pals‐22*(*jy3*) mutants (PALS‐22 is a negative regulator of the IPR) (Figure 4b and Table S8). These genes include *lys‐7*, *osg‐1*, *kin‐5*, *skr‐5*, and *dod‐19* (Figure 4c). Hence, HSF‐1 may inhibit their expression.

To determine the reason why HSF‐1 deficiency differentially affects thermotolerance in 1‐ versus 4‐day‐old adults, we examined the expression of several genes by qPCR in both wild‐type and *hsf‐1*(*sy441*) mutant animals under physiological vs. heat shock‐induced conditions. Genes were selected that showed increased expression in 1‐day‐old adults and were related to stress‐ or innate immune response. The analysis uncovered that UPR^ER^‐related genes, including *lips‐11*, *calu‐1*, and *warf‐1*, are expressed at higher levels in the *hsf‐1*(*sy441*) mutant background than in control (Figure 4d–f). The expression of genes involved in innate immunity, such as *pals‐6*, *lys‐7*, and *skr‐5*, was also enhanced in 1‐day‐old *hsf‐1*(*sy441*) mutant adults, but this difference was abolished at the stage of 4 days of adulthood (Figure 4g–i). Genes related to various stress response pathways such as IlS (*sod‐3* and *asp‐8*) and SKN‐1‐mediated oxidative stress response (*osg‐1*) displayed similar expression patterns during aging in *hsf‐1*(*sy441*) mutant and wild‐type nematodes (Figure 4j–l). All these data indicate that the induction of stress‐ and innate immunity‐related genes in the *hsf‐1*(*sy441*) mutant background depends on the age of animals.

### 2.6 The UPR^ER^ pathway and SKN‐1 are required for the increased thermotolerance of HSF‐1 depleted animals

To understand why thermotolerance in *hsf‐1*(*RNAi*) nematodes at the stage of early (1‐day‐old) adulthood becomes increased relative to wild type, we hypothesized that HSF‐1 deficiency causes a modest cellular stress, thereby inducing the activity of distinct cellular stress response pathways. This change eventually leads to increased heat resistance in nematodes with reduced HSF‐1 activity. To test this idea, we first checked whether the expression of stress response reporters is induced in the *hsf‐1*(*sy441*) mutant background.

IlS is a conserved cellular stress response pathway that can be activated by high temperatures (Lithgow et al., 1995; McColl et al., 2010). Decreased activity of the pathway results in elevated thermotolerance. We tested the activity of IlS using a *sod‐3p::gfp* reporter, *muls84*. *sod‐3* encodes a superoxide dismutase that is a well‐described target of DAF‐16/FOXO, the terminal transcription factor of the IlS pathway (Honda & Honda, 1999). We observed a significant increase in *sod‐3::gfp* expression in animals with reduced HSF‐1 activity [*hsf‐1*(*sy441*) mutants and *hsf‐1*(*RNAi*) animals] as compared to the wild‐type, suggesting that IlS activity is decreased when HSF‐1 becomes compromised (Figure S3a,b). Inactivation of DAF‐16/FOXO is known to suppress the elevated thermotolerance of mutant animals with lowered IlS activity (McColl et al., 2010). We observed that the increased thermotolerance of *hsf‐1*(*sy441*) mutants was not suppressed by silencing *daf‐16* (Figure S3c).Similarly, downregulation of HSF‐1 increases the thermotolerance of *daf‐16*(*m26*) mutant animals to the same extent found in the wild‐type background (Figure S3d). Thus, the thermal resistance of 1‐day‐old adults depleted for HSF‐1 is independent of DAF‐16/FOXO activity.

Autophagy, a highly conserved, lysosome‐mediated degradation process of eukaryotic cells, is also required for the maintenance of proteostasis (Aman et al., 2021; Sigmond et al., 2008; Sigmond & Vellai, 2023; Vellai, 2021; Vellai et al., 2009). We tested whether expression of the autophagy marker *mCherry::gfp::lgg‐1* (Chang et al., 2017) is altered in the *hsf‐1*(*RNAi*) background, but found no significant change (Figure S4a–c). In line with this observation, silencing three autophagy‐related (*atg*) genes, *atg‐7*, *atg‐18* and *lgg‐1*, did not suppress increased thermotolerance in *hsf‐1*(*sy441*) mutant animals at the early adult stages (Figure S4d). Similar results were obtained when analysing the thermotolerance of *atg‐18*(*gk378*); *hsf‐1*(*sy441*) and *epg‐7*(*tm2508*); *hsf‐1*(*sy441*) double mutant animals (Figure S4e,f). These results suggest that the elevated thermotolerance of 1‐day‐old animals with decreased HSF‐1 activity is not mediated by autophagy either.

**FIGURE 5:**
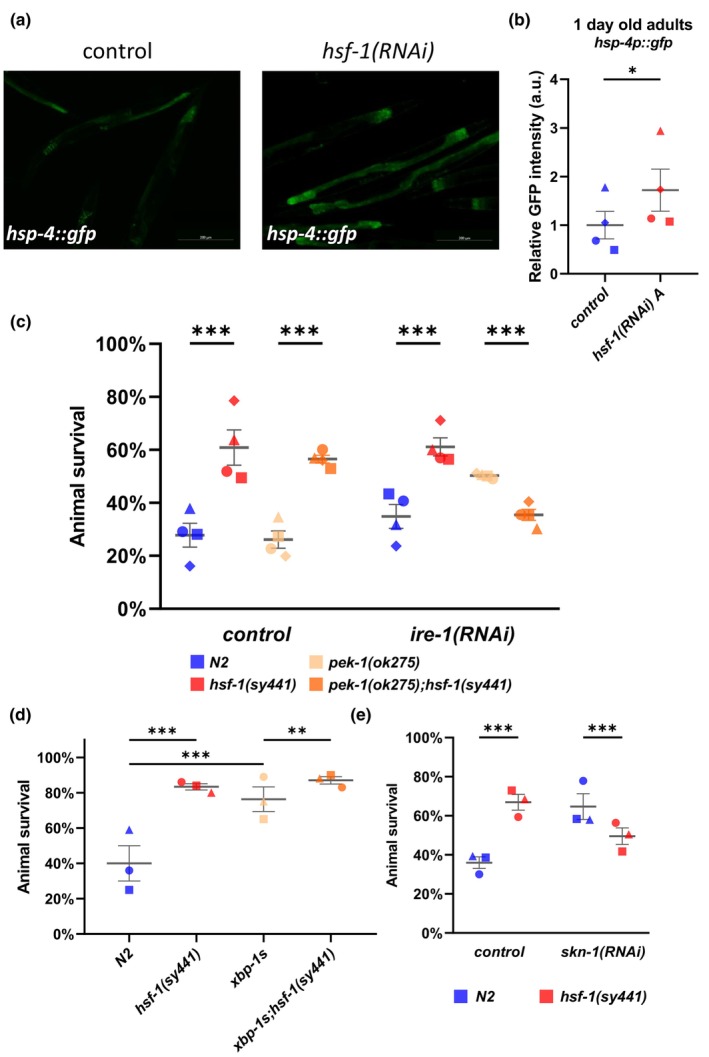
Inactivation of the UPR^ER^ and SKN‐1 suppresses the increased thermotolerance of *hsf‐1* mutant animals. (a) The expression of the *hsp‐4p::gfp* marker is increased when *hsf‐1* is silenced in 1 day old adult *C. elegans* (representative figure). (b) Quantified expression of *hsp‐4p::gfp* reporter in wild‐type and *hsf‐1*(*RNAi*) genetic backgrounds. Paired *t*‐test, * = *p* < 0.05 ** = *p* < 0.01 *** = *p* < 0.001; error bars represent ± SEM. (c) Inactivating two (IRE‐1‐ and PEK‐1‐mediated) of the three signaling arms of the UPR^ER^ suppressed the increased thermotolerance of the 1 day old adult *hsf‐1*(*sy441*) *C. elegans* (animals were shifted from 20°C to 35°C for 6 h). (d) Increased XBP‐1 activity significantly elevated heat stress tolerance of both wild‐type and hsf‐1(sy441) mutant animals (animals were shifted from 20°C to 35°C for 6 h). (e) The increased thermotolerance of the *hsf‐1*(*sy441*) animals is suppressed in the *skn‐1*(*RNAi*) background (animals were shifted from 20°C to 35°C for 6 h). Cochran–Mantel–Haenszel test; * = *p* < 0.05 ** = *p* < 0.01 *** = *p* < 0.001; Data points represent mean of independent trials (the different biological replicates are indicated by different shapes), lines represent mean of means, error bars represent ± SEM. Source data underlying Figure 5b–e are provided in Table S13.

Besides the HSR, the UPR^ER^ is also required for proteostasis in the ER (Taylor & Hetz, 2020). So, it is possible that increased activity of the UPR^ER^ contributes to thermoresistance in HSF‐1‐depleted animals. To address this issue, we analyzed the expression of a marker protein of UPR, HSP‐4, the ortholog of the Hsp70 family protein BiP/HSPA5, which is a key chaperone of the ER. We measured the expression of an *hsp‐4p::gfp* reporter (*zcIs4*) in wild‐type and *hsf‐1*(*RNAi*) genetic backgrounds and found that the expression is significantly elevated when HSF‐1 activity is depleted (Figure 5a,b). It is intriguing that at the adult stage of 2 and 4 days, such an increase in *hsp‐4p::gfp* expression was not detectable (Figure S5d,e).

The UPR^ER^ can be activated by three parallel intracellular signaling pathways; the first is mediated by IRE‐1α/XBP‐1 s (inositol‐requiring protein 1α/spliced X box‐binding protein 1) proteins, the second involves PERK/ATF‐4 (protein kinase RNA‐like endoplasmic reticulum kinase/activating transcription factor 4) proteins, while the third relies on ATF‐6/ATF‐6f (activating transcription factor 6/cytosolic domain fragment of ATF‐6) proteins (Hetz, 2012). We showed that the thermotolerance of *hsf‐1*(*sy441*); *pek‐1*(*ok275*) double mutants is increased compared to *pek‐1*(*ok275*) single mutant animals (Figure 5c). This suggests that inactivation of a single (e.g., IRE‐1α/XBP‐1 s) branch of the UPR^ER^ cannot suppress the increased thermotolerance caused by decreased HSF‐1 activity. In contrast, we detected a significant suppression of thermotolerance in *hsf‐1*(*sy441*) mutant animals when two branches of the UPR^ER^ were simultaneously depleted in the *pek‐1*(*ok275*); *ire‐1*(*RNAi*) double defective genetic background (Figure 5c). Moreover, similar effects were observed in *ire‐1*(*ok799*); *atf‐6*(*RNAi*) and *ire‐1*(*ok799*); *pek‐1*(*RNAi*) genetic backgrounds as well (Figure S5a,b), indicating that the UPR^ER^ is required for the hormesis induced by the *hsf‐1*(*sy441*) mutation. Of note, we observed a minor difference in the thermotolerance of *ire‐1*(*ok799*) single mutants as compared to *ire‐1*(*ok799*); *hsf‐1*(*sy441*) double mutant animals (Figure S5a,b). These data suggest that the absence of IRE‐1 activity per se slightly suppresses the increased thermotolerance of *hsf‐1*(*sy441*) mutants. We also analyzed whether *xbp‐1* alone influences the thermotolerance of *hsf‐1*(*sy441*) mutant animals. According to our results, *xbp‐1* downregulation alone is not sufficient to suppress elevated thermotolerance in *hsf‐1*(*sy441*) mutants (Figure S5c). To support the fact that increased activity of the UPR^ER^ pathway enhances thermotolerance in 1‐day‐old adult nematodes, we analyzed the stress tolerance of animals expressing *xbp‐1 s*, which encodes for a constitutively active form of XBP‐1 (Imanikia et al., 2019). These results showed that increased XBP‐1 activity significantly enhances heat stress tolerance in both wild‐type and *hsf‐1*(*sy441*) mutant animals. However, XBP‐1 hyperactivity in *hsf‐1*(*sy441*) mutant background causes only a slight increase in thermotolerance, suggesting that XBP‐1 hyperactivity and HSF‐1 deficiency may act in the same pathway (Figure 5d).

It was shown recently that the proteasome subunit gene *rpt‐2* is upregulated by SKN‐1A in hypomorphic *hsf‐1*(*sy441*) mutant animals (Lehrbach & Ruvkun, 2019). SKN‐1A is an ER‐associated isoform that translocates from the ER lumen to the cytoplasm by the ER‐associated degradation (ERAD) machinery, and enters the nucleus where it can upregulate target genes upon impaired proteosome function (Lehrbach & Ruvkun, 2016). We found that a common set of genes is upregulated in *hsf‐1*(*sy441*) mutant and *skn‐1*(*RNAi*) genetic backgrounds (Figure 2c,e). This raises the possibility that SKN‐1 activity contributes to the increased thermotolerance of *hsf‐1*(*sy441*) mutant animals at the early adult stages. We thus determined whether downregulation of *skn‐1* may influence thermotolerance in *hsf‐1*(*sy441*) mutant worms (Figure 5e). In line with recent observations (Deng et al., 2020; Frankino et al., 2022), *skn‐1*(*RNAi*) animals were found to tolerate high temperatures significantly better than control, but this effect was suppressed in the *hsf‐1*(*sy441*); *skn‐1*(*RNAi*) genetic background, suggesting a mutual compensatory interaction between the conserved stress response pathways mediated by SKN‐1/Nrf2 and HSF‐1.

### 2.7 The innate immunity‐related signalling system contributes to increased thermotolerance at the first day of adulthood

**FIGURE 6:**
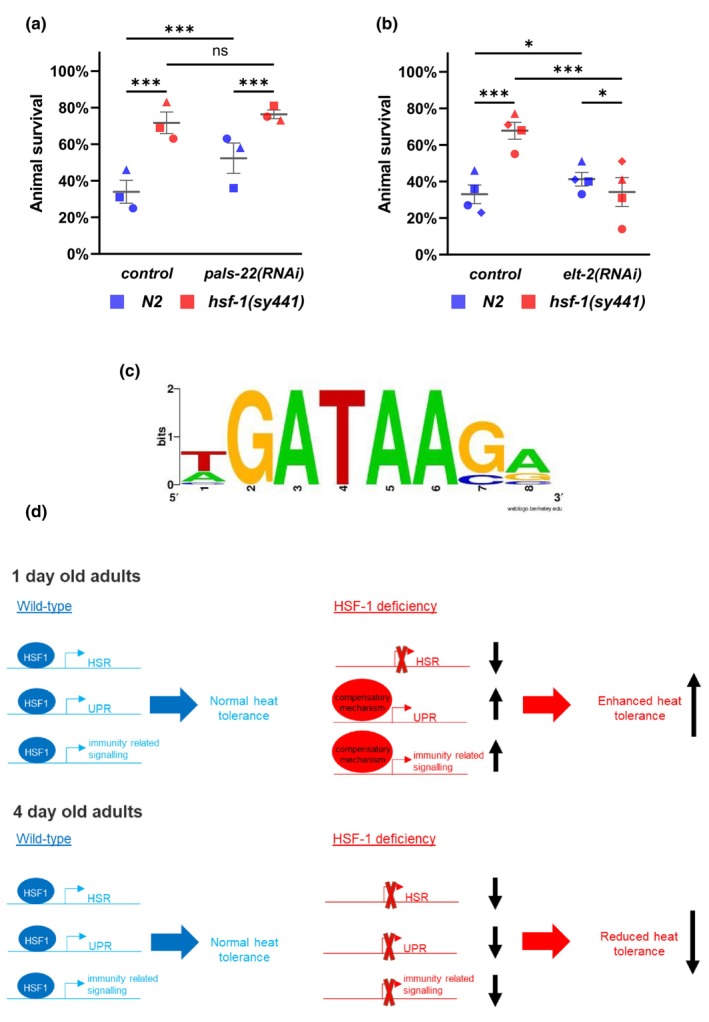
Immunity related signaling contributes to the increased thermotolerance of *hsf‐1* mutant animals. (a) Silencing *pals‐22* increases heat tolerance to the same extent in wild type and *hsf‐1*(*sy441*) genetic backgrounds (animals were shifted from 20°C to 35°C for 6 h). (b) Silencing *elt‐2* suppresses the increased thermotolerance of *hsf‐1*(*sy441*) mutants (animals were shifted from 20°C to 35°C for 6 h). Cochran–Mantel–Haenszel test; * = *p* < 0.05 ** = *p* < 0.01 *** = *p* < 0.001; Data points represent mean of independent trials (the different biological replicates are indicated by different shapes), lines represent mean of means, error bars represent ± SEM. (c) An ELT‐2 binding site related motif is common in genes upregulated in *hsf‐1*(*sy441*) mutant and *hsf‐1*(*RNAi*) background. Enrichment ratio: 2.94 compared to motif found in randomly generated sequences; FRD:3.17E‐08 (d) Our current model: HSF‐1 deficiency induces a mild proteotoxic stress, as a result, activity of other cellular stress response pathways – mainly the unfolded protein response (UPR^ER^)and immunity related signaling – is elevated in young animals; paradoxically this hormetic effect contributes to a heat resistant phenotype of 1 day old HSF1 deficient *C. elegans*. Source data underlying Figure 6a,b are provided in Table S15.

Since genes functioning in the innate immune response were significantly enriched in gene sets upregulated in both *hsf‐1*(*sy441*) mutant and *hsf‐1*(*RNAi*) animals, we tested whether the response contributes to enhanced heat stress tolerance in *hsf‐1*(*RNAi*) nematodes at the first day of adulthood. Recently, it was shown that PALS‐22 deficiency leads to increased thermotolerance mediated by a cullin‐RING ubiquitin ligase, a component of the intracellular pathogen response in *C. elegans* (Panek et al., 2020). We found that silencing *pals‐22* increased thermotolerance in wild‐type animals but not in *hsf‐1*(*sy441*) mutant background, suggesting that the IPR and HSF‐1 deficiency act in the same pathway to enhance thermotolerance (Figure 6a).

ELT‐2, a GATA transcription factor, has been reported to be essential for the development of the intestine (McGhee et al., 2009). The protein also regulates the expression of host defense genes (Kerry et al., 2006). We performed a thermotolerance assay to test whether *elt‐2* and *hsf‐1* interact in regulating thermotolerance. The results indicate that the elevated heat stress tolerance of 1‐day‐old *hsf‐1*(*sy441*) mutant adults is suppressed by silencing *elt‐2* (Figure 6b). We further found that a conserved ELT‐2 binding site‐related motif is enriched in the promoter of genes commonly upregulated in *hsf‐1*(*sy441*) mutant and *hsf‐1*(*RNAi*) genetic backgrounds (Figure 6c and Table S16). Together, the innate immunity‐related signaling system is required for the enhanced stress tolerance of *hsf‐1*(*sy441*) mutant animals.

## 3 DISCUSSION

In this work, we showed that decreased activity of HSF‐1, the master regulator of the HSR, results in increased thermotolerance in *C. elegans* at the first day of adulthood (Figure 1b,c). We also found that the effect of reduced HSF‐1 activity on thermotolerance changes with age. The enhanced stress tolerance triggered by reduced HSF‐1 activity is obvious at the first day of adulthood but is rapidly abolished as the animal grows older. Comparing RNAseq data we obtained with published gene expression datasets showed that the UPR^ER^ and innate immunity‐associated genes, as well as genes downregulated by SKN‐1/Nrf2, are induced in 1‐day‐old *hsf‐1*(*sy441*) mutant adults (Figure 2b, Table S5). A qPCR analysis supported that induction of these stress response‐ and innate immunity‐related genes is abolished in older nematodes deficient in HSF‐1 function (Figure 4d–l). Based on these data we constructed a model according to which the elevated heat tolerance of 1‐day‐old adults is due to the elevated activity of genes involved in innate immunity and the UPR^ER^. However, inducibility of stress response in *C. elegans* markedly decreases with age (De‐Souza et al., 2022; Dues et al., 2016; Taylor & Dillin, 2013). This result offers an explanation for an age‐dependent hormesis in animals with reduced HSF‐1 activity; in young adults, induction of the UPR^ER^ and innate immunity‐related genes may compensate the decreased activity of the HSR pathway, but in older animals, the pathway becomes less active (Figure 6d).

The age‐dependent role of HSF‐1 in thermotolerance may also explain why so many contradictory data are available in the literature on heat stress tolerance in *hsf‐1*(*sy441*) mutant animals. Several publications showed that 2‐day‐old *hsf‐1*(*sy441*) mutant adults tolerate high temperatures with the same extent than wild type (Dues et al., 2016; Kourtis et al., 2012; McColl et al., 2010). Moreover, others reported results (Morton, 2013) that are highly similar to those being reported in our present study. Another research group found that downregulating *hsf‐1* also increases the heat stress tolerance (Golden et al., 2020). These results seemingly contradict with the paradigm that HSF‐1 is a master regulator of the heat stress tolerance by activating the expression of HSPs. However, it has been shown that overexpressing the C‐terminally truncated form of HSF‐1 (HSF‐1ΔTAD) increases heat stress tolerance in transgenic animals, but the expression of hsp genes remains unchanged under physiological conditions (Baird et al., 2014). This suggests that HSF‐1‐mediated activation of genes encoding non‐HSPs also plays a key role in heat stress tolerance. This study also shows that overexpression of HSF‐1ΔTAD enhances thermotolerance and inhibits aging by upregulating *pat‐10*, which in turn helps to maintain the integrity of the actin cytoskeleton (Baird et al., 2014). Another study supported this finding showing that HSF‐1 is essential for regulating cytoskeletal integrity during aging (Higuchi‐Sanabria, Paul, et al., 2018). Although our present analysis shows that genes involved in muscle cell development and actomyosin structure organization are overrepresented among the genes upregulated in *hsf‐1*(*sy441*) mutant animals (Figure 2b), we did not identify *pat‐10* as a differentially expressed gene in *hsf‐1*(*sy441*) mutants (Table S4). We also found that *hsf‐1* transcript levels are not altered in *hsf‐1*(*sy441*) mutants as compared to wild type (Table S4). These results indicate that overexpression of truncated HSF‐1 and *hsf‐1* deficiency enhance heat stress tolerance through different pathways.

Our present work shows that the expression of several genes encoding HSPs is increased in the *hsf‐1*(*sy441*) hypomorphic mutant background after heat shock (Figure 3). *sy441* allele determines a truncated protein that lacks a significant part of the predicted transcriptional activation domain (Hajdu‐Cronin et al., 2004). However, as our results above suggest, the truncated protein is still able to activate genes encoding HSPs upon heat stress. Consistent with this finding, it has been shown that increased expression of *hsp‐70b* (*F44E5.4*) gene upon heat shock was not affected by the *hsf‐1*(*sy441*) mutation (Chisnell et al., 2018). According to our RNAseq and qPCR analyses, induction of *hsp‐70b* was even enhanced in the *hsf‐*(*sy441*) mutant background (Figure 3e). Moreover, an RNAseq analysis demonstrates that, although at reduced levels compared to the wild‐type, several HSPs are induced when only truncated HSF‐1 (HSF‐1 ΔTAD) is expressed (Baird et al., 2014).

It is an intriguing question how a truncated protein with a defective transactivation domain can activate gene transcription. It may be speculated that the induction of HSPs in *hsf‐1*(*sy441*) mutant animals is not due to the activity of the truncated protein. However, our result showing that heat shock‐induced activation of *hsp‐16.2p::gfp* reporter is suppressed in the *hsf‐1*(*sy441*); *hsf‐1*(*RNAi*) double defective genetic background indicating that transcription of HSP‐encoding genes is mediated by HSF‐1ΔTAD (Figure 3h,i). Thus, the C‐terminally truncated transcription factor can retain some of its activity. Of particular importance, we highlight our finding that the expression of a *hsp‐16p::gfp* reporter is increased exclusively in the intestine of *hsf‐1*(*sy441*) mutant animals exposed to heat shock (Figure 3h). Such a tissue‐specific activity of HSF‐1 needs further investigation, but it is conceivable that truncated HSF‐1 preserves its activity by interacting with other proteins expressed exclusively in the gut. The further identification of such interactors would be of major interest for the field.

Our GSEA analysis showed that genes upregulated in both *hsf‐1*(*sy441*) mutant and *hsf‐1*(*RNAi*) backgrounds showed a significant overlap with genes implicated in innate immunity (Figure 2c). A significant set of these genes functions in the intracellular pathogen response regulated by *pals‐22* and *pals‐25* (Reddy et al., 2019). Interestingly, it has been reported that inactivating *pal‐22* induces the IPR, and this leads to enhanced thermotolerance through the upregulation of a cullin‐RING ubiquitin ligase complex (Panek et al., 2020). Several components of the complex, such as *skr‐4* and *skr‐5* (Skps), and several F‐box proteins (*fbxa‐163* and *fbxa‐58*), are upregulated in *pals‐22*(*jy3*) and *hsf‐1*(*sy441*) mutants and in the *hsf‐1*(*RNAi*) genetic background. These data, along with present our result showing that increased thermotolerance caused by depleting either *pals‐22* or *hsf‐1* is not additive (Figure 6a), indicate that these two pathways may act together in regulating heat stress tolerance. A close interaction between *pals‐22* and *hsf‐1* is also supported by the fact that *pals‐22*(*jy3*); *hsf‐1*(*sy441*) double mutants have been reported to exhibit larval lethality (Reddy et al., 2017).

ELT‐2 is thought to activate innate immune response against gut pathogens (Yang et al., 2016). Our present results show that ELT‐2 activity is required for enhanced thermotolerance of 1‐day‐old *hsf‐1*(*sy441*) mutant animals (Figure 6b). This finding, together with the presence of conserved GATA transcription factor binding sites in the promoter of genes activated upon HSF‐1 depletion, suggest that innate immunity‐related genes are activated by ELT‐2, and this contributes to increased thermotolerance.

Our data presented in this work also indicate that the UPR^ER^ is required for enhanced thermotolerance in young *hsf‐1*(*sy441*) mutant animals (Figure 5 and Figure S5). However, it is intriguing how reduced HSF‐1 activity can lead to the activation of the UPR^ER^. Recently, it has been shown in mice that the HSF‐1‐β‐catenin signaling axis inhibits XBP‐1 and activates innate immune response in IR‐stressed liver inflammation in macrophages (Yue et al., 2016). Crosstalk between HSF‐1 and the UPR has been described earlier. For example, inhibiting overall translation through eIF4G/IFG‐1 has been shown to enhance the ER and cytoplasmic proteostasis through a mechanism that is dependent of HSF‐1 activity (Howard et al., 2016). It has been reported that impairing SIRT‐1 and HSF‐1 leads to a B12‐dependent ER stress in oleosin‐transcobalamin chimera (OT) cells, suggesting an interaction between cytoplasmic and ER‐specific UPR (Ghemrawi et al., 2013). Finally, it is conceivable that interaction between HSF‐1 and the UPR^ER^ is mediated by the induction of innate immunity genes upon *hsf‐1* deficiency. Several proteins playing a role in host defense are synthesized in the ER and secreted into the extracellular space, thereby overloading the ER and causing ER stress (Richardson et al., 2010). This offers an alternative explanation why inactivation of the UPR^ER^ also suppresses the enhanced thermotolerance of HSF‐1‐deficient worms. The role of the UPR^ER^ in this process was further supported by showing that hyperactivation of the UPR^ER^ using a constitutively active form of XBP‐1 (Imanikia et al., 2019) results in increased thermotolerance as compared to control in both wild‐type and *hsf‐1*(*sy441*) mutant backgrounds (Figure 5d).

Interaction between cellular stress responses, innate immune response, and the extracellular proteostasis machinery has been elucidated (Gallotta et al., 2020; Jung et al., 2023). It has been shown that the IPR functions in maintaining extracellular proteostasis and that inactivation of *hsf‐1* ameliorates the age‐dependent decline in extracellular proteostasis, possibly by inducing the expression of IPR‐related genes (Jung et al., 2023). Of note, we have found that some genes that are upregulated in the *hsf‐1*(*sy441*) mutant background as compared to wild‐type code for proteins that have been reported to function as extracellular chaperones (Gallotta et al., 2020) (Table S16).

According to our present model, 1‐day‐old adult nematodes with lowered HSF‐1 activity tolerate high temperatures better than wild‐type, most likely as a result of a compensatory effect mediated by other stress response pathways (Figure 6d). Indeed, cellular stress response systems including, for example, the HSR, UPR, and autophagy do not operate separately but are interconnected. There are many examples where knocking out one stress response pathway leads to the overactivation of another one. In mammalian cells, for example, proteasome inhibitors can significantly increase the expression of cytosolic and ER‐resident chaperones and confer increased heat tolerance (Bush et al., 1997; Khan et al., 2012; Pirkkala et al., 2000; Young & Heikkila, 2010). In *Drosophila*, disruption of proteasomal degradation has been shown to result in increased autophagic activity (Lőw et al., 2013). Moreover, in yeast, inactivation of key chaperones of different compartments induces a uniform cell‐wide stress response that increases replicative and chronological life span through activation of both metabolic and proteostatic genes (Perić et al., 2016). It has also been shown that constitutive SKN‐1 activation impairs thermotolerance, while silencing *skn‐1* results in increased heat resistance (Deng et al., 2020; Frankino et al., 2022). These data together support that mild stress resulting from the reduced function of the HSR may increase the activity of other stress response pathways, leading to enhanced thermotolerance.

Cellular stress response pathways, such as the Nrf2‐mediated oxidative stress response pathway, UPR^ER^ are critical for maintaining proteostasis, and loss of proteostasis is a feature that characterizes essentially all aging cells (López‐Otín et al., 2023; Zhang et al., 2022). Imbalances in these molecular machineries promote senescence and lead to accelerated aging. In this study, we presented that depletion of HSF‐1 activity induces the SKN‐1/Nrf2‐mediated and innate immunity‐related systems, together with the UPRER, controlling tolerance to heat stress in *C. elegans*. Furthermore, this effect of HSF‐1 deficiency manifests in an age‐dependent manner. Hence, results demonstrated in this work may lead to a deeper insight into the mechanisms by which these conserved molecular pathways contribute to life span determination.

## 4 CONCLUDING REMARKS

In this work, we showed that decreased activity of HSF‐1, the master regulator of the HSR in *C. elegans*, leads to increased heat stress tolerance at early adult stages. This seemingly illogical phenomenon is not so surprising when one considers that under natural conditions animals are constantly confronted with changing environmental factors where different stressors occur at different times and to different degrees. In response to stress, a rapid and then a rapidly decaying response has been evolved to enhance survival of the animal. However, when the activity of a master regulator of stress responses is genetically altered (hyperactivated or inhibited), the outcome may be difficult to predict (Lamech & Haynes, 2015). As HSF‐1 is a promising therapeutical target in cancer treatment, it is also particularly worth considering that a similarly artificial situation may exist during medical treatments.

## 5 EXPERIMENTAL PROCEDURES

### 5.1 *C. elegans* strains and maintenance

Unless otherwise indicated, nematodes were maintained and propagated at 20°C on nematode growth medium (NGM)‐containing plates and fed with *Escherichia coli* OP50 bacteria. The *C. elegans* strains used in this study can be found in Table S18.

### 5.2 RNA sequencing

Wild‐type and *hsf‐1*(*sy441*) mutant nematodes were synchronized and stored at 20°C until they have reached the young adult stage. Worms were then heat‐shocked at 35°C for 30 min, and after a 1‐h long recovery at 20°C, RNA was isolated from heat‐treated versus untreated worms. Total RNA was prepared using RNAzol® RT (MRC, RN 190) standard protocols and then cleaned up on Rneasy columns (QIAgen, cat# 74104). Gel electrophoresis using a 1% agarose gel were performed for a visual determination of sample quality. RNA integrity analysis, sample preparation, and RNA sequencing was performed by Novogene on an Illumina NovaSeq PE150 platform. A quality‐control analysis of raw RNA‐seq reads was performed by using the FastQC program. Calculation of log_2_(fold change), *p* values and FDR (corrected *p* values) was performed by EdgeR using Galaxy (Afgan et al., 2018). WBCel235 was used as the reference genome for annotation. Venny 2.1 was used to construct Venn diagrams for determining HSF‐1‐regulated transcripts (Oliveros, 2015). Statistical over‐representation tests of gene sets were performed using the PANTHER database (http://PANTHERdb.org, Accessed on 15 January 2024) (Mi et al., 2021). To analyze statistical significance, Fisher's exact test with Benjamini–Hochberg False Discovery Rate correction (FDR) was applied. RNA‐seq reads were uploaded to the NCBI GEO database with Accession number GSE241011.

### 5.3 Quantitative real‐time PCR

RNA was isolated using RNAzol® RT (RN 190) (Molecular Research Center, INC.; 5645 Montgomery Road, Cincinnati, OH 45212, USA) and purified from the aqueous phase after extraction using RNA Clean & ConcentratorTM‐5 kit (R1013) (Zymo Research Co.; 17,062 Murphy Ave., Irvine, CA 92614, USA). cDNA was synthesized using the RevertAid First Strand cDNA Synthesis Kit (K1622) (Thermo Fisher Scientific Inc.; 81 Wyman St., Waltham, MA 02451, USA).

Quantitative real‐time PCR was performed using the Roche LightCycler® 96 System (F. Hoffmann‐La Roche AG, Grenzacherstrasse 124, 4070 Basel, Switzerland), with Maxima SYBR Green/ROX qPCR Master Mix (2X) (K0222) (Thermo Fisher Scientific Inc.; 81 Wyman St, Waltham, MA 02451, USA).

For the primers that were used, see Table S18.

### 5.4 Gene set enrichment and Venn diagram analysis

For GSEA analyses, the list of upregulated genes in the *hsf‐1*(*sy441*) mutants (this study) and in *hsf‐1*(*RNAi*) background (Brunquell et al., 2016) was used. GSEA was performed using GSEA v3.0 software applying the Preranked module (Subramanian et al., 2005). DEGs were ranked from highest to lowest based on Log2 fold changes, and converted into a GSEA‐compatible file. The gene sets used for comparison are based on a published gene set collection (Reddy et al., 2019), modified by adding additional gene sets, and converted into a GSEA‐compatible file. GSEA was performed using a signal‐to‐noise metric of 1000 permutations with ‘no collapse’. Results were graphed according to their NES value using GraphPad Prism 7.

The gene lists described above were used for the Venn diagram analysis. Venny 2.1 was used to construct Venn diagrams. Representation factors and significance of overlaps were determined using nemates.org: for ‘total number of genes’ we used 20,570, which is the size of our RNA‐seq dataset after filtering out low‐count and undetected genes.

### 5.5 Motif analysis

Promoter region motif enrichment analysis was done on the promoter regions 1000 bp upstream to translation start sites of the genes upregulated in *hsf‐1*(*sy441*) mutant and *hsf‐1*(*RNAi*) background using MEME suite (Bailey et al., 2015). The consensus sequence was visualized using WebLogo (Crooks et al., 2004).

### 5.6 RNA interference

RNA was isolated from a mixed‐age population of wild‐type *C. elegans* strain, using RNAzol® RT (RN 190) (Molecular Research Center, Inc.; 5645 Montgomery Road, Cincinnati, OH 45212, USA). Using isolated RNA as template, cDNA was synthesized by the RevertAid First Strand cDNA Synthesis Kit (K1622) (Thermo Fisher Scientific Inc.; 81 Wyman St., Waltham, MA 02451, USA). To generate *C. elegans* RNAi clones, 600–1000 base pair‐long cDNA fragments were amplified by PCR, using cDNA as template and cloned into the vector L4440 (Addgene; plasmid #1654). In case of *pals‐22*, TEDA cloning was applied (Xia et al., 2019). RNAi constructs were transformed into *E. coli* HT115(DE3) used as food source. For primers, see Table S10. The RNAi construct against *skn‐1* was a gift from T. Keith Blackwell (Harvard Medical School, Boston MA, USA). The RNAi construct against *daf‐16* was the same as the one used in (Hotzi et al., 2018). In case of *hsf‐1* RNAi constructs, a cDNA clone of *hsf‐1* (*yk610c7*, gift of Yuji Kohara) was subcloned into L4440, using restriction enzymes HindIII and KpnI (for *hsf‐1* RNAi A). In case of *hsf‐1* RNAi, a BamHI and PstI fragment of *yk610c7* was cloned into T444T, an improved RNAi vector that contains two T7 transcription termination sequences to ensure the production of specific double‐stranded RNA (Sturm et al., 2018, 2023). *hsf‐1* silencing was validated by testing the induction of *hsp‐16.2p::gfp* (*gpIs1*) upon heat shock in control and *hsf‐1*(*RNAi*) animals. Worms were fed from hatch with *E. coli* HT115 strain containing an empty vector (control) or expressing double‐stranded RNA.

### 5.7 Thermotolerance assay

Five to 10 gravid adults were allowed to lay eggs for 4–6 h at 20°C to obtain a synchronous population. In case of *ire‐1*(*ok799*), *ire‐1*(*ok799*); *hsf‐1*(*sy441*), *atg‐18*(*gk378*) and *atg‐18*(*gk378*); *hsf‐1*(*sy441*) mutants, due to delayed growth, worms were synchronized 12–24 h prior to the other strains in order to obtain 1‐day‐old adults at the day of thermotolerance measurement. Synchronized, 1‐day‐old adult worms were placed at 35°C for 5 h on NGM plates seeded with OP50 *E. coli*. In case of RNAi experiments, nematodes were heat‐shocked for 6 h as after 5 h at 35°C, the survival of the worms raised on HT115 bacteria transformed with empty vector L4440 or the appropriate RNAi construct was almost 100% (Figure S1c). Worms were then placed at 20°C, and after 16 h, they were scored for viability. At least 90 worms were used per category on three technical parallel plates, and experiments were repeated at least three times.

For pre‐heat shock, 18 h prior to the thermotolerance assay, worms were placed at 35°C for 30 min, and then placed at 20°C until the assay.

To perform thermotolerance assays, at the onset of egg laying, the synchronization of worms was carried out 62, 64, and 71 h before the assay: 10 gravid adults were allowed to lay eggs for 1 h at 20°C.

In case of determining the thermotolerance of 2‐ and 4‐day‐old worms, to bypass the egg‐laying defect of *hsf‐1*‐depleted worms, FUdR was used in 0.025 mg/mL concentration to make worms sterile.

In case of thermotolerance assays for aged, 2‐ and 4‐day‐old animals, when FUdR was not supplemented, we reared the worms on normal NGM plates and carefully selected those animals that did not show an egg‐laying phenotype for the assay.

To perform ‘online’ thermotolerance assays, synchronized worms were maintained at 20°C until the young adult stage, then transferred at 35°C to NGM seeded with *E. coli* OP50 bacteria or HT115 containing either empty L4440 feeding vector (EV) or a fragment of *hsf‐1* cDNA *hsf‐1*(*RNAi*). Approximately 20–30 young (non‐gravid) adults were transferred to the assay plates. Animals were scored hourly and considered dead when they stopped pharyngeal pumping and responding to touching. SPSS 17 software was used to calculate mean lifespan and perform statistical analysis. The *p* values for comparing Kaplan–Meier survival curves between two groups were determined using log‐rank (Mantel‐Cox) tests.

### 5.8 Quantification of fluorophore expression intensity

For fluorescence microscopy, worms were immobilized using 100 mM Sodium‐azide, and images were captured with Zeiss AXIO Imager.M2 epifluorescence microscopes with a given exposure time. Measurements were performed with the Image J software. The fluorescent intensity of selected areas was calculated by subtracting the mean grey value of the background from the mean grey value of the object of interest (the same size of areas was selected). We evaluated reporter intensity in at least three independent experiments. In one experiment, we analyzed at least 10 animals and estimated the fluorophore intensity based on the average of the intensities observed in these animals. The final value and SEM were then calculated as the average of the means of the independent experiments.

### 5.9 Statistical analysis

Statistical significance for all assays was determined using RStudio (Version 3.4.3), GraphPad Prism (GraphPad Software Inc., San Diego, CA, USA), and IBM SPSS Statistics (International Business Machines Corporation, Armonk, NY, USA) statistical softwares. Statistical significance is demarcated in figures as *p < 0.05, **p < 0.01, and ***p < 0.001.

## AUTHOR CONTRIBUTIONS

J.B.B‐ and J.B. invented the project. D.K., J.B.B. and J.B. conceived the experiments. D.K. and J.B. analyzed the data and wrote the manuscript. D.K., J.B.B, M.K., S.A., V.V.V., U.M. and J.B. generated strains, performed thermotolerance assays. M.K. and D.K. isolated RNA and performed qPCR experiments. V.V.V., D.K., U.M. and S.A. designed and generated RNAi constructs. D.K., T.S., B.H., and J.B. performed imaging experiments. D.K. and J.B. prepared samples for RNA sequencing experiments. M.V. and J.B. analyzed the results of RNA sequencing experiments. B.H., M.V. and J.B. performed GSEA and Venn analysis. J.B., D.K., J.B.B., B.H., S.A., T.S., and T.V. provided conceptual feedback on the manuscript and edited the manuscript. T.V. provided financial support for the project.

## CONFLICT OF INTEREST STATEMENT

The authors declare no competing financial interest.

## Supporting information


Figure S1.

Figure S2.

Figure S3.

Figure S4.

Figure S5.

Table S1.

Table S2.

Table S3.

Table S4.

Table S5.

Table S6.

Table S7.

Table S8.

Table S9.

Table S10.

Table S11.

Table S12.

Table S13.

Table S14.

Table S15.

Table S16.

Table S17.

Table S18.

## Data Availability

The data that supports the findings of this study are available in the supplementary material of this article.

## References

[acel14246-bib-0001] Afgan, E. , Baker, D. , Batut, B. , van den Beek, M. , Bouvier, D. , Cech, M. , Chilton, J. , Clements, D. , Coraor, N. , Grüning, B. A. , Guerler, A. , Hillman‐Jackson, J. , Hiltemann, S. , Jalili, V. , Rasche, H. , Soranzo, N. , Goecks, J. , Taylor, J. , Nekrutenko, A. , & Blankenberg, D. (2018). The galaxy platform for accessible, reproducible and collaborative biomedical analyses: 2018 update. Nucleic Acids Research, 46, W537–W544. 29790989 10.1093/nar/gky379 PMC6030816

[acel14246-bib-0002] Akerfelt, M. , Morimoto, R. I. , & Sistonen, L. (2010). Heat shock factors: Integrators of cell stress, development and lifespan. Nature Reviews. Molecular Cell Biology, 11, 545–555. 20628411 10.1038/nrm2938 PMC3402356

[acel14246-bib-0003] Aman, Y. , Schmauck‐Medina, T. , Hansen, M. , Morimoto, R. I. , Simon, A. K. , Bjedov, I. , Palikaras, K. , Simonsen, A. , Johansen, T. , Tavernarakis, N. , Rubinsztein, D. C. , Partridge, L. , Kroemer, G. , Labbadia, J. , & Fang, E. F. (2021). Autophagy in healthy aging and disease. Nature Aging, 1, 634–650. 34901876 10.1038/s43587-021-00098-4 PMC8659158

[acel14246-bib-0004] Bailey, T. L. , Johnson, J. , Grant, C. E. , & Noble, W. S. (2015). The MEME suite. Nucleic Acids Research, 43, W39–W49. 25953851 10.1093/nar/gkv416 PMC4489269

[acel14246-bib-0005] Baird, N. A. , Douglas, P. M. , Simic, M. S. , Grant, A. R. , Moresco, J. J. , Wolff, S. C. , Yates, J. R. , Manning, G. , & Dillin, A. (2014). HSF‐1‐mediated cytoskeletal integrity determines thermotolerance and life span. Science, 346, 360–363. 25324391 10.1126/science.1253168 PMC4403873

[acel14246-bib-0006] Barna, J. , Csermely, P. , & Vellai, T. (2018). Roles of heat shock factor 1 beyond the heat shock response. Cellular and Molecular Life Sciences, 75, 2897–2916. 29774376 10.1007/s00018-018-2836-6 PMC11105406

[acel14246-bib-0007] Barna, J. , Princz, A. , Kosztelnik, M. , Hargitai, B. , Takács‐Vellai, K. , & Vellai, T. (2012). Heat shock factor‐1 intertwines insulin/IGF‐1, TGF‐β and cGMP signaling to control development and aging. BMC Developmental Biology, 12, 32. 23116063 10.1186/1471-213X-12-32 PMC3558376

[acel14246-bib-0008] Bar‐Ziv, R. , Frakes, A. E. , Higuchi‐Sanabria, R. , Bolas, T. , Frankino, P. A. , Gildea, H. K. , Metcalf, M. G. , & Dillin, A. (2020). Measurements of physiological stress responses in C. *elegans* . Journal of Visualized Experiments, 159, 61001. 10.3791/61001 PMC7840273 32510480

[acel14246-bib-0009] Brunquell, J. , Morris, S. , Lu, Y. , Cheng, F. , & Westerheide, S. D. (2016). The genome‐wide role of HSF‐1 in the regulation of gene expression in *Caenorhabditis elegans* . BMC Genomics, 17, 559. 27496166 10.1186/s12864-016-2837-5 PMC4975890

[acel14246-bib-0010] Bush, K. T. , Goldberg, A. L. , & Nigam, S. K. (1997). Proteasome inhibition leads to a heat‐shock response, induction of endoplasmic reticulum chaperones, and thermotolerance. The Journal of Biological Chemistry, 272, 9086–9092. 9083035 10.1074/jbc.272.14.9086

[acel14246-bib-0011] Chang, J. T. , Kumsta, C. , Hellman, A. B. , Adams, L. M. , & Hansen, M. (2017). Spatiotemporal regulation of autophagy during *Caenorhabditis elegans* aging. eLife, 6, e18459. 28675140 10.7554/eLife.18459 PMC5496740

[acel14246-bib-0012] Chisnell, P. , Parenteau, T. R. , Tank, E. , Ashrafi, K. , & Kenyon, C. (2018). The mTOR target S6 kinase arrests development in *Caenorhabditis elegans* when the heat‐shock transcription factor is impaired. Genetics, 210, 999–1009. 30228197 10.1534/genetics.118.301533 PMC6218238

[acel14246-bib-0013] Crooks, G. E. , Hon, G. , Chandonia, J. M. , & Brenner, S. E. (2004). WebLogo: a sequence logo generator. Genome Research, 14, 1188–1190. 15173120 10.1101/gr.849004 PMC419797

[acel14246-bib-0014] Dai, C. , & Sampson, S. B. (2016). HSF1: Guardian of proteostasis in cancer. Trends in Cell Biology, 26, 17–28. 26597576 10.1016/j.tcb.2015.10.011 PMC4722819

[acel14246-bib-0015] Deng, J. , Dai, Y. , Tang, H. , & Pang, S. (2020). SKN‐1 is a negative regulator of DAF‐16 and somatic stress resistance in *Caenorhabditis elegans* . G3: Genes, Genomes, Genetics, 10, 1707–1712. 32161088 10.1534/g3.120.401203 PMC7202003

[acel14246-bib-0016] De‐Souza, E. A. , Cummins, N. , & Taylor, R. C. (2022). IRE‐1 endoribonuclease activity declines early in C. elegans adulthood and is not rescued by reduced reproduction. Frontiers in Aging, 3, 1044556. 36389122 10.3389/fragi.2022.1044556 PMC9649906

[acel14246-bib-0017] Dong, B. , Jaeger, A. M. , & Thiele, D. J. (2019). Inhibiting heat shock factor 1 in cancer: A unique therapeutic opportunity. Trends in Pharmacological Sciences, 40, 986–1005. 31727393 10.1016/j.tips.2019.10.008

[acel14246-bib-0018] Dues, D. J. , Andrews, E. K. , Schaar, C. E. , Bergsma, A. L. , Senchuk, M. M. , & Van Raamsdonk, J. M. (2016). Aging causes decreased resistance to multiple stresses and a failure to activate specific stress response pathways. Aging (Albany NY), 8, 777–795. 27053445 10.18632/aging.100939 PMC4925828

[acel14246-bib-0019] Finger, F. , Ottens, F. , & Hoppe, T. (2021). The Argonaute Proteins ALG‐1 and ALG‐2 are Linked to Stress Resistance and Proteostasis (Vol. 2021). Micropublication Biology. 10.17912/micropub.biology.000457 PMC8553546 34723149

[acel14246-bib-0020] Frankino, P. A. , Siddiqi, T. F. , Bolas, T. , Bar‐Ziv, R. , Gildea, H. K. , Zhang, H. , Higuchi‐Sanabria, R. , & Dillin, A. (2022). SKN‐1 regulates stress resistance downstream of amino catabolism pathways. iScience, 25, 104571. 35784796 10.1016/j.isci.2022.104571 PMC9240870

[acel14246-bib-0021] Gallotta, I. , Sandhu, A. , Peters, M. , Haslbeck, M. , Jung, R. , Agilkaya, S. , Blersch, J. L. , Rödelsperger, C. , Röseler, W. , Huang, C. , Sommer, R. J. , & David, D. C. (2020). Extracellular proteostasis prevents aggregation during pathogenic attack. Nature, 584, 410–414. 32641833 10.1038/s41586-020-2461-z

[acel14246-bib-0022] Ghemrawi, R. , Pooya, S. , Lorentz, S. , Gauchotte, G. , Arnold, C. , Gueant, J. L. , & Battaglia‐Hsu, S. F. (2013). Decreased vitamin B12 availability induces ER stress through impaired SIRT1‐deacetylation of HSF1. Cell Death & Disease, 4, e553. 23519122 10.1038/cddis.2013.69 PMC3615730

[acel14246-bib-0023] Golden, N. L. , Plagens, R. N. , Kim Guisbert, K. S. , & Guisbert, E. (2020). Standardized methods for measuring induction of the heat shock response in *Caenorhabditis elegans* . Journal of Visualized Experiments, 161, 61030. 10.3791/61030 32716378

[acel14246-bib-0024] Hajdu‐Cronin, Y. M. , Chen, W. J. , & Sternberg, P. W. (2004). The L‐type cyclin CYL‐1 and the heat‐shock‐factor HSF‐1 are required for heat‐shock‐induced protein expression in *Caenorhabditis elegans* . Genetics, 168, 1937–1949. 15611166 10.1534/genetics.104.028423 PMC1448743

[acel14246-bib-0025] Hetz, C. (2012). The unfolded protein response: Controlling cell fate decisions under ER stress and beyond. Nature Reviews. Molecular Cell Biology, 13, 89–102. 22251901 10.1038/nrm3270

[acel14246-bib-0026] Higuchi‐Sanabria, R. , Frankino, P. A. , Paul, J. W. , Tronnes, S. U. , & Dillin, A. (2018). A futile battle? Protein quality control and the stress of aging. Developmental Cell, 44, 139–163. 29401418 10.1016/j.devcel.2017.12.020 PMC5896312

[acel14246-bib-0027] Higuchi‐Sanabria, R. , Paul, J. W. , Durieux, J. , Benitez, C. , Frankino, P. A. , Tronnes, S. U. , Garcia, G. , Daniele, J. R. , Monshietehadi, S. , & Dillin, A. (2018). Spatial regulation of the Actin cytoskeleton by HSF‐1 during aging. Molecular Biology of the Cell, 29, 2522–2527. 30133343 10.1091/mbc.E18-06-0362 PMC6254583

[acel14246-bib-0028] Hipp, M. S. , Kasturi, P. , & Hartl, F. U. (2019). The proteostasis network and its decline in ageing. Nature Reviews. Molecular Cell Biology, 20, 421–435. 30733602 10.1038/s41580-019-0101-y

[acel14246-bib-0029] Honda, Y. , & Honda, S. (1999). The daf‐2 gene network for longevity regulates oxidative stress resistance and Mn‐superoxide dismutase gene expression in *Caenorhabditis elegans* . The FASEB Journal, 13, 1385–1393. 10428762

[acel14246-bib-0030] Hotzi, B. , Kosztelnik, M. , Hargitai, B. , Takács‐Vellai, K. , Barna, J. , Bördén, K. , Málnási‐Csizmadia, A. , Lippai, M. , Ortutay, C. , Bacquet, C. , Pasparaki, A. , Arányi, T. , Tavernarakis, N. , & Vellai, T. (2018). Sex‐specific regulation of aging in *Caenorhabditis elegans* . Aging Cell, 17, e12724. 29493066 10.1111/acel.12724 PMC5946081

[acel14246-bib-0031] Howard, A. C. , Rollins, J. , Snow, S. , Castor, S. , & Rogers, A. N. (2016). Reducing translation through eIF4G/IFG‐1 improves survival under ER stress that depends on heat shock factor HSF‐1 in *Caenorhabditis elegans* . Aging Cell, 15, 1027–1038. 27538368 10.1111/acel.12516 PMC5114698

[acel14246-bib-0032] Hsu, A.‐L. , Murphy, C. T. , & Kenyon, C. (2003). Regulation of aging and age‐related disease by DAF‐16 and heat‐shock factor. Science, 300, 1142–1145. 12750521 10.1126/science.1083701

[acel14246-bib-0033] Imanikia, S. , Özbey, N. P. , Krueger, C. , Casanueva, M. O. , & Taylor, R. C. (2019). Neuronal XBP‐1 activates intestinal lysosomes to improve Proteostasis in *C*. *elegans* . Current Biology, 29, 2322–2338.e7. 31303493 10.1016/j.cub.2019.06.031 PMC6658570

[acel14246-bib-0034] Joutsen, J. , & Sistonen, L. (2019). Tailoring of Proteostasis networks with heat shock factors. Cold Spring Harbor Perspectives in Biology, 11, a034066. 30420555 10.1101/cshperspect.a034066 PMC6442201

[acel14246-bib-0035] Jung, R. , Lechler, M. C. , Fernandez‐Villegas, A. , Chung, C. W. , Jones, H. C. , Choi, Y. H. , Thompson, M. A. , Rödelsperger, C. , Röseler, W. , Kaminski Schierle, G. S. , Sommer, R. J. , & David, D. C. (2023). A safety mechanism enables tissue‐specific resistance to protein aggregation during aging in *C*. *elegans* . PLoS Biology, 21, e3002284. 37708127 10.1371/journal.pbio.3002284 PMC10501630

[acel14246-bib-0036] Kerry, S. , TeKippe, M. , Gaddis, N. C. , & Aballay, A. (2006). GATA transcription factor required for immunity to bacterial and fungal pathogens. PLoS One, 1, e77. 17183709 10.1371/journal.pone.0000077 PMC1762309

[acel14246-bib-0037] Khan, S. , Rammeloo, A. W. , & Heikkila, J. J. (2012). Withaferin a induces proteasome inhibition, endoplasmic reticulum stress, the heat shock response and acquisition of thermotolerance. PLoS One, 7, e50547. 23226310 10.1371/journal.pone.0050547 PMC3511540

[acel14246-bib-0038] Kourtis, N. , Nikoletopoulou, V. , & Tavernarakis, N. (2012). Small heat‐shock proteins protect from heat‐stroke‐associated neurodegeneration. Nature, 490, 213–218. 22972192 10.1038/nature11417

[acel14246-bib-0039] Kourtis, N. , & Tavernarakis, N. (2011). Cellular stress response pathways and ageing: Intricate molecular relationships. The EMBO Journal, 30, 2520–2531. 21587205 10.1038/emboj.2011.162 PMC3155297

[acel14246-bib-0040] Kovács, D. , Kovács, M. , Ahmed, S. , & Barna, J. (2022). Functional diversification of heat shock factors. Biologia Futura, 73, 427–439. 36402935 10.1007/s42977-022-00138-z

[acel14246-bib-0041] Kyriakou, E. , Taouktsi, E. , & Syntichaki, P. (2022). The thermal stress coping network of the nematode *Caenorhabditis elegans* . International Journal of Molecular Sciences, 23, 14907. 36499234 10.3390/ijms232314907 PMC9737000

[acel14246-bib-0042] Labbadia, J. , & Morimoto, R. I. (2015). Repression of the heat shock response is a programmed event at the onset of reproduction. Molecular Cell, 59, 639–650. 26212459 10.1016/j.molcel.2015.06.027 PMC4546525

[acel14246-bib-0043] Lamech, L. T. , & Haynes, C. M. (2015). The unpredictability of prolonged activation of stress response pathways. The Journal of Cell Biology, 209, 781–787. 26101215 10.1083/jcb.201503107 PMC4477854

[acel14246-bib-0044] Lehrbach, N. J. , & Ruvkun, G. (2016). Proteasome dysfunction triggers activation of SKN‐1A/Nrf1 by the aspartic protease DDI‐1. eLife, 5, e17721. 27528192 10.7554/eLife.17721 PMC4987142

[acel14246-bib-0045] Lehrbach, N. J. , & Ruvkun, G. (2019). Endoplasmic reticulum‐associated SKN‐1A/Nrf1 mediates a cytoplasmic unfolded protein response and promotes longevity. eLife, 8, e44425. 30973820 10.7554/eLife.44425 PMC6459674

[acel14246-bib-0046] Li, J. , Labbadia, J. , & Morimoto, R. I. (2017). Rethinking HSF1 in stress, development, and organismal health. Trends in Cell Biology, 27, 895–905. 28890254 10.1016/j.tcb.2017.08.002 PMC5696061

[acel14246-bib-0047] Lithgow, G. J. , White, T. M. , Melov, S. , & Johnson, T. E. (1995). Thermotolerance and extended life‐span conferred by single‐gene mutations and induced by thermal stress. Proceedings of the National Academy of Sciences, 92, 7540–7544. 10.1073/pnas.92.16.7540 PMC41375 7638227

[acel14246-bib-0048] López‐Otín, C. , Blasco, M. A. , Partridge, L. , Serrano, M. , & Kroemer, G. (2023). Hallmarks of aging: An expanding universe. Cell, 186, 243–278. 36599349 10.1016/j.cell.2022.11.001

[acel14246-bib-0049] Lőw, P. , Varga, Á. , Pircs, K. , Nagy, P. , Szatmári, Z. , Sass, M. , & Juhász, G. (2013). Impaired proteasomal degradation enhances autophagy via hypoxia signaling in drosophila. BMC Cell Biology, 14, 29. 23800266 10.1186/1471-2121-14-29 PMC3700814

[acel14246-bib-0050] McColl, G. , Rogers, A. N. , Alavez, S. , Hubbard, A. E. , Melov, S. , Link, C. D. , Bush, A. I. , Kapahi, P. , & Lithgow, G. J. (2010). Insulin‐like signaling determines survival during stress via posttranscriptional mechanisms in *C. elegans* . Cell Metabolism, 12, 260–272. 20816092 10.1016/j.cmet.2010.08.004 PMC2945254

[acel14246-bib-0051] McGhee, J. D. , Fukushige, T. , Krause, M. W. , Minnema, S. E. , Goszczynski, B. , Gaudet, J. , Kohara, Y. , Bossinger, O. , Zhao, Y. , Khattra, J. , Hirst, M. , Jones, S. J. M. , Marra, M. A. , Ruzanov, P. , Warner, A. , Zapf, R. , Moerman, D. G. , & Kalb, J. M. (2009). ELT‐2 is the predominant transcription factor controlling differentiation and function of the *C. elegans* intestine, from embryo to adult. Developmental Biology, 327, 551–565. 19111532 10.1016/j.ydbio.2008.11.034 PMC2706090

[acel14246-bib-0052] Mi, H. , Ebert, D. , Muruganujan, A. , Mills, C. , Albou, L.‐P. , Mushayamaha, T. , & Thomas, P. D. (2021). PANTHER version 16: A revised family classification, tree‐based classification tool, enhancer regions and extensive API. Nucleic Acids Research, 49, D394–D403. 33290554 10.1093/nar/gkaa1106 PMC7778891

[acel14246-bib-0053] Morimoto, R. I. (2020). Cell‐nonautonomous regulation of Proteostasis in aging and disease. Cold Spring Harbor Perspectives in Biology, 12, a034074. 30962274 10.1101/cshperspect.a034074 PMC7111247

[acel14246-bib-0054] Morton, E. (2013). Regulation and dynamic behavior of the heat shock transcription factor Hsf‐1 in *C. elegans* . In Doctoral Dissertation. University of Pennsylvania.

[acel14246-bib-0055] Morton, E. A. , & Lamitina, T. (2013). *Caenorhabditis elegans* HSF‐1 is an essential nuclear protein that forms stress granule‐like structures following heat shock. Aging Cell, 12, 112–120. 23107491 10.1111/acel.12024 PMC3552056

[acel14246-bib-0056] Oliveros, J. C. (2015). Venny 2.1. 0. An interactive tool for comparing lists with Venn's diagrams. BioinfoGP of CNB‐CSIC .

[acel14246-bib-0057] Panek, J. , Gang, S. S. , Reddy, K. C. , Luallen, R. J. , Fulzele, A. , Bennett, E. J. , & Troemel, E. R. (2020). A cullin‐RING ubiquitin ligase promotes thermotolerance as part of the intracellular pathogen response in *Caenorhabditis elegans* . Proceedings of the National Academy of Sciences, 117, 7950–7960. 10.1073/pnas.1918417117 PMC7148578 32193347

[acel14246-bib-0058] Perić, M. , Bou Dib, P. , Dennerlein, S. , Musa, M. , Rudan, M. , Lovrić, A. , Nikolić, A. , Šarić, A. , Sobočanec, S. , Mačak, Ž. , Raimundo, N. , & Kriško, A. (2016). Crosstalk between cellular compartments protects against proteotoxicity and extends lifespan. Scientific Reports, 6, 28751. 27346163 10.1038/srep28751 PMC4921836

[acel14246-bib-0059] Pirkkala, L. , Alastalo, T. P. , Zuo, X. , Benjamin, I. J. , & Sistonen, L. (2000). Disruption of heat shock factor 1 reveals an essential role in the ubiquitin proteolytic pathway. Molecular and Cellular Biology, 20, 2670–2675. 10733569 10.1128/mcb.20.8.2670-2675.2000 PMC85482

[acel14246-bib-0060] Prahlad, V. , Cornelius, T. , & Morimoto, R. I. (2008). Regulation of the cellular heat shock response in *Caenorhabditis elegans* by thermosensory neurons. Science, 320, 811–814. 18467592 10.1126/science.1156093 PMC3429343

[acel14246-bib-0061] Reddy, K. C. , Dror, T. , Sowa, J. N. , Panek, J. , Chen, K. , Lim, E. S. , Wang, D. , & Troemel, E. R. (2017). An intracellular pathogen response pathway promotes proteostasis in *C. elegans* . Current Biology, 27, 3544–3553.e5. 29103937 10.1016/j.cub.2017.10.009 PMC5698132

[acel14246-bib-0062] Reddy, K. C. , Dror, T. , Underwood, R. S. , Osman, G. A. , Elder, C. R. , Desjardins, C. A. , Cuomo, C. A. , Barkoulas, M. , & Troemel, E. R. (2019). Antagonistic paralogs control a switch between growth and pathogen resistance in *C. elegans* . PLoS Pathogens, 15, e1007528. 30640956 10.1371/journal.ppat.1007528 PMC6347328

[acel14246-bib-0063] Revtovich, A. V. , Lee, R. , & Kirienko, N. V. (2019). Interplay between mitochondria and diet mediates pathogen and stress resistance in *Caenorhabditis elegans* . PLoS Genetics, 15, e1008011. 30865620 10.1371/journal.pgen.1008011 PMC6415812

[acel14246-bib-0064] Richardson, C. E. , Kooistra, T. , & Kim, D. H. (2010). An essential role for XBP‐1 in host protection against immune activation in *C. elegans* . Nature, 463, 1092–1095. 20182512 10.1038/nature08762 PMC2834299

[acel14246-bib-0065] Roos‐Mattjus, P. , & Sistonen, L. (2021). Interplay between mammalian heat shock factors 1 and 2 in physiology and pathology. The FEBS Journal, 289, 7710–7725. 34478606 10.1111/febs.16178

[acel14246-bib-0066] Shen, X. , Ellis, R. E. , Sakaki, K. , & Kaufman, R. J. (2005). Genetic interactions due to constitutive and inducible gene regulation mediated by the unfolded protein response in *C. elegans* . PLoS Genetics, 1, e37. 16184190 10.1371/journal.pgen.0010037 PMC1231716

[acel14246-bib-0067] Sigmond, T. , Barna, J. , Tóth, M. L. , & Takács‐Vellai, K. (2008). Autophagy in *Caenorhabditis elegans* . Methods in Enzymology, 451, 521–540. 19185738 10.1016/S0076-6879(08)03230-8

[acel14246-bib-0068] Sigmond, T. , & Vellai, T. (2023). Lysosomal alteration links food limitation to longevity. Nature Aging, 3, 1048–1050. 37620583 10.1038/s43587-023-00483-1

[acel14246-bib-0069] Somogyvári, M. , Khatatneh, S. , & Sőti, C. (2022). Hsp90: From cellular to organismal proteostasis. Cells, 11, 2479. 36010556 10.3390/cells11162479 PMC9406713

[acel14246-bib-0070] Steinkraus, K. A. , Smith, E. D. , Davis, C. , Carr, D. , Pendergrass, W. R. , Sutphin, G. L. , Kennedy, B. K. , & Kaeberlein, M. (2008). Dietary restriction suppresses proteotoxicity and enhances longevity by an hsf‐1‐dependent mechanism in *Caenorhabditis elegans* . Aging Cell, 7, 394–404. 18331616 10.1111/j.1474-9726.2008.00385.x PMC2709959

[acel14246-bib-0071] Sturm, Á. , Saskői, É. , Hotzi, B. , Tarnóci, A. , Barna, J. , Bodnár, F. , Sharma, H. , Kovács, T. , Ari, E. , Weinhardt, N. , Kerepesi, C. , Perczel, A. , Ivics, Z. , & Vellai, T. (2023). Downregulation of transposable elements extends lifespan in *Caenorhabditis elegans* . Nature Communications, 14, 5278. 10.1038/s41467-023-40957-9 PMC10465613 37644049

[acel14246-bib-0072] Sturm, Á. , Saskoi, É. , Tibor, K. , Weinhardt, N. , & Vellai, T. (2018). Highly efficient RNAi and Cas9‐based auto‐cloning systems for *C. elegans* research. Nucleic Acids Research, 46, e105. 29924347 10.1093/nar/gky516 PMC6158509

[acel14246-bib-0073] Subramanian, A. , Tamayo, P. , Mootha, V. K. , Mukherjee, S. , Ebert, B. L. , Gillette, M. A. , Paulovich, A. , Pomeroy, S. L. , Golub, T. R. , Lander, E. S. , & Mesirov, J. P. (2005). Gene set enrichment analysis: A knowledge‐based approach for interpreting genome‐wide expression profiles. Proceedings of the National Academy of Sciences, 102, 15545–15550. 10.1073/pnas.0506580102 PMC1239896 16199517

[acel14246-bib-0074] Sutphin, G. L. , & Kaeberlein, M. (2009). Measuring *Caenorhabditis elegans* life span on solid media. Journal of Visualized Experiments, 27, e1152. 10.3791/1152 PMC2794294 19488025

[acel14246-bib-0075] Taylor, R. C. , & Dillin, A. (2013). XBP‐1 is a cell‐nonautonomous regulator of stress resistance and longevity. Cell, 153, 1435–1447. 23791175 10.1016/j.cell.2013.05.042 PMC4771415

[acel14246-bib-0076] Taylor, R. C. , & Hetz, C. (2020). Mastering organismal aging through the endoplasmic reticulum proteostasis network. Aging Cell, 19, e13265. 33128506 10.1111/acel.13265 PMC7681052

[acel14246-bib-0077] Vellai, T. (2021). How the amino acid leucine activates the key cell‐growth regulator mTOR. Nature, 596, 192–194. 34290413 10.1038/d41586-021-01943-7

[acel14246-bib-0078] Vellai, T. , Takács‐Vellai, K. , Sass, M. , & Klionsky, D. J. (2009). The regulation of aging: Does autophagy underlie longevity? Trends in Cell Biology, 19, 487–494. 19726187 10.1016/j.tcb.2009.07.007 PMC2755611

[acel14246-bib-0079] Whitesell, L. , & Lindquist, S. (2009). Inhibiting the transcription factor HSF1 as an anticancer strategy. Expert Opinion on Therapeutic Targets, 13, 469–478. 19335068 10.1517/14728220902832697

[acel14246-bib-0080] Xia, Y. , Li, K. , Li, J. , Wang, T. , Gu, L. , & Xun, L. (2019). T5 exonuclease‐dependent assembly offers a low‐cost method for efficient cloning and site‐directed mutagenesis. Nucleic Acids Research, 47, e15. 30462336 10.1093/nar/gky1169 PMC6379645

[acel14246-bib-0081] Yang, W. , Dierking, K. , Rosenstiel, P. C. , & Schulenburg, H. (2016). GATA transcription factor as a likely key regulator of the *Caenorhabditis elegans* innate immune response against gut pathogens. Zoology (Jena, Germany), 119, 244–253. 27372411 10.1016/j.zool.2016.05.013

[acel14246-bib-0082] Young, J. T. F. , & Heikkila, J. J. (2010). Proteasome inhibition induces hsp30 and hsp70 gene expression as well as the acquisition of thermotolerance in Xenopus laevis A6 cells. Cell Stress & Chaperones, 15, 323–334. 19838833 10.1007/s12192-009-0147-4 PMC2866991

[acel14246-bib-0083] Yue, S. , Zhu, J. , Zhang, M. , Li, C. , Zhou, X. , Zhou, M. , Ke, M. , Busuttil, R. W. , Ying, Q. , Kupiec‐Weglinski, J. W. , Xia, Q. , & Ke, B. (2016). The myeloid HSF1‐Β‐catenin Axis regulates NLRP3 Inflammasome activation in mouse liver ischemia/reperfusion injury. Hepatology, 64, 1683–1698. 27474884 10.1002/hep.28739 PMC5074868

[acel14246-bib-0084] Zevian, S. C. , & Yanowitz, J. L. (2014). Methodological considerations for heat shock of the nematode *Caenorhabditis elegans* . Methods, 68, 450–457. 24780523 10.1016/j.ymeth.2014.04.015 PMC4112136

[acel14246-bib-0085] Zhang, W. H. , Koyuncu, S. , & Vilchez, D. (2022). Insights into the links between proteostasis and aging from *C. elegans* . Frontiers in Aging, 3, 854157. 35821832 10.3389/fragi.2022.854157 PMC9261386

