# Supplementary figures and images for: Age‐dependent heat shock hormesis to HSF‐1 deficiency suggests a compensatory mechanism mediated by the unfolded protein response and innate immunity in young Caenorhabditis elegans
Source: Aging Cell. 2024 Jun 19;23(10):e14246. doi: 10.1111/acel.14246 (PMC11464127; doi:10.1111/acel.14246)

**A**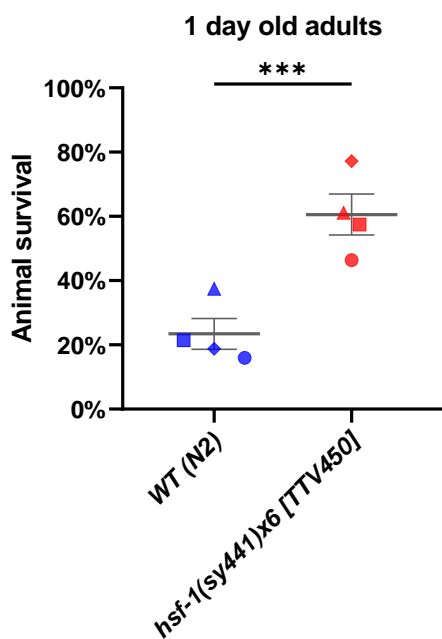**B**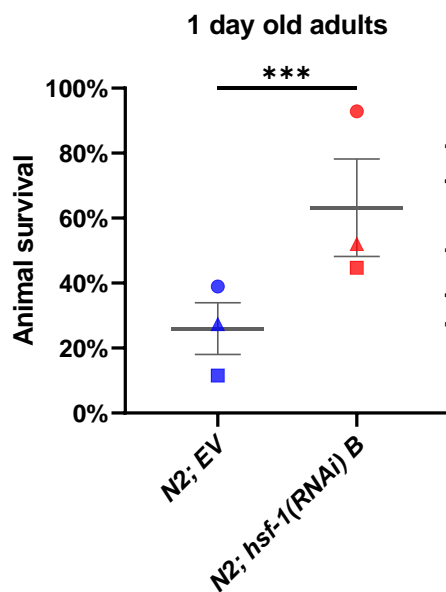**C**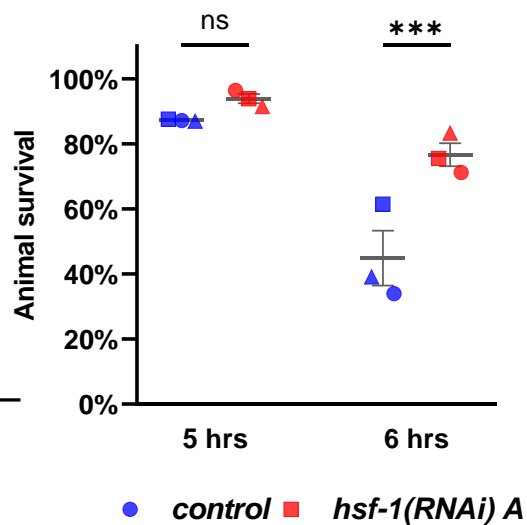**D**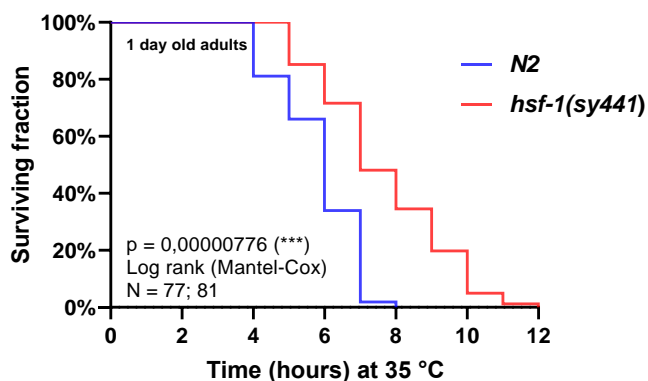**E**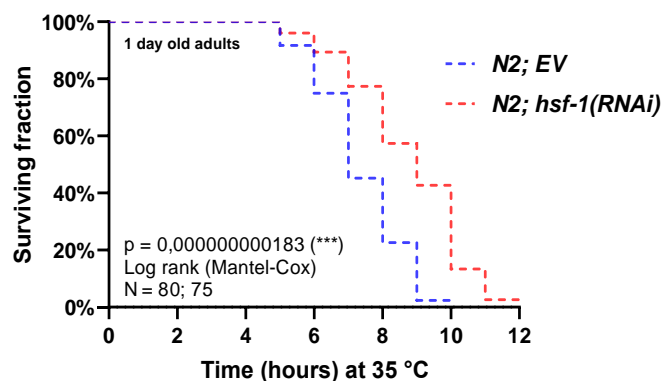**F**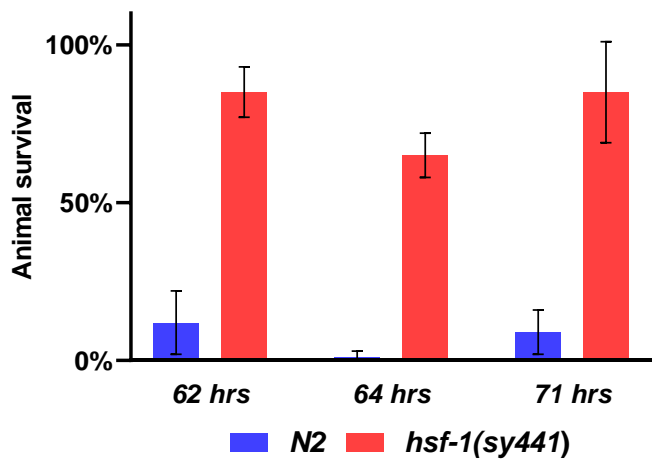**G**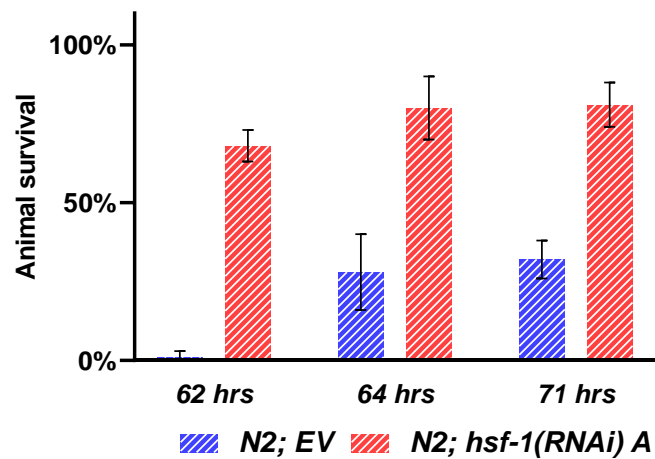

Supplement: Supplementary file 1 — Figure S1. [file ACEL-23-e14246-s003.pdf]

**A**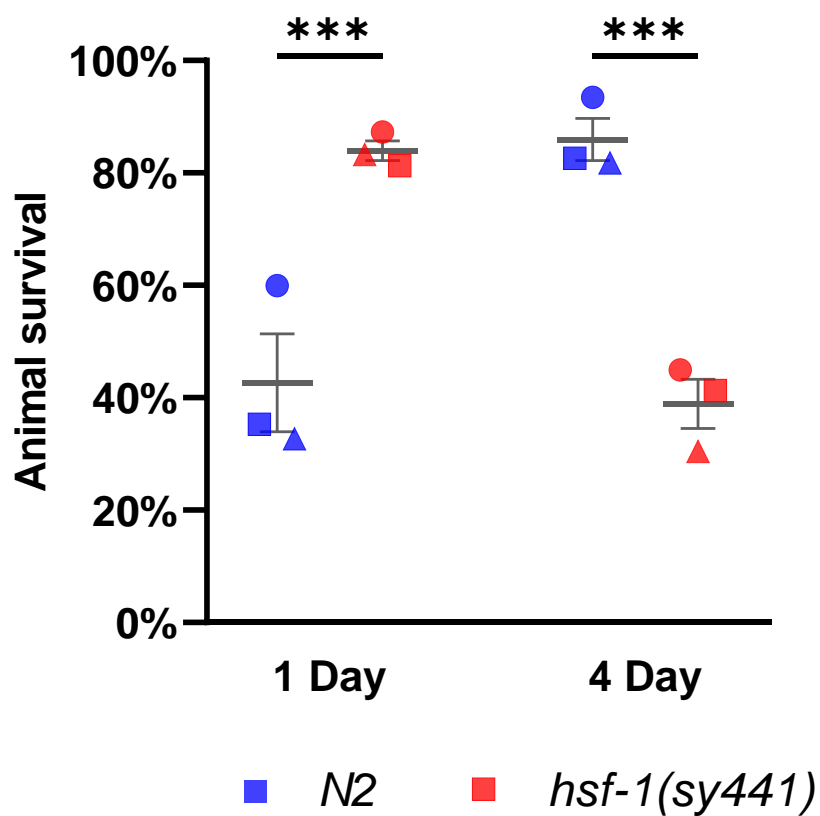**B**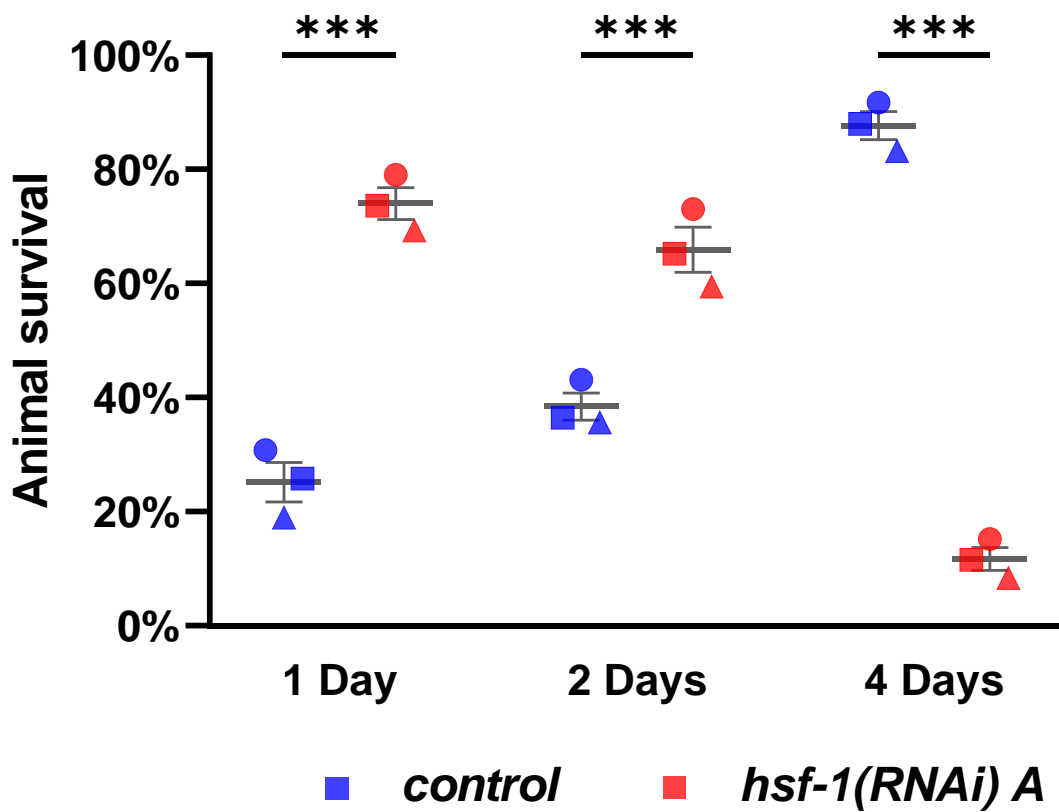

Supplement: Supplementary file 2 — Figure S2. [file ACEL-23-e14246-s001.pdf]

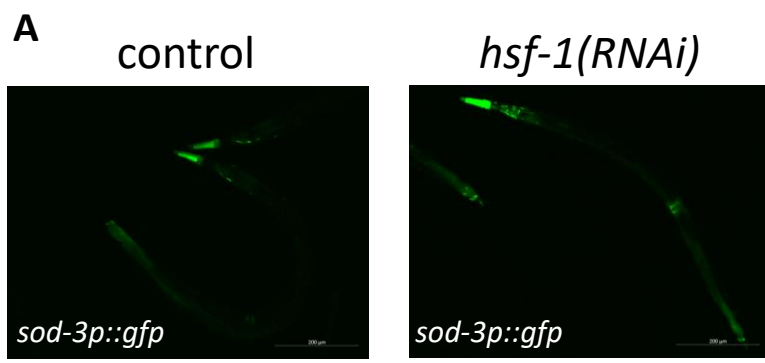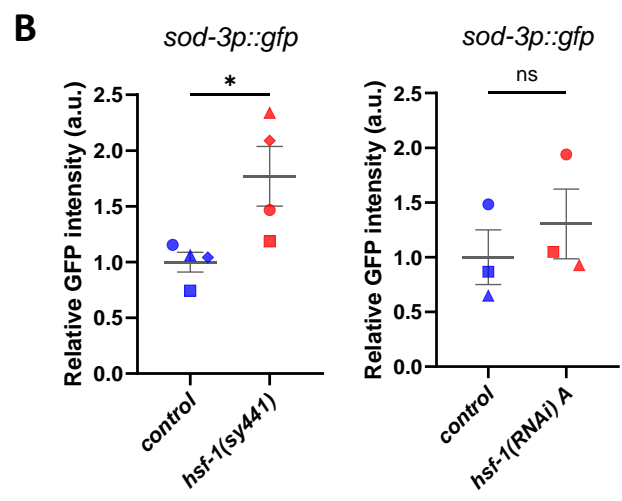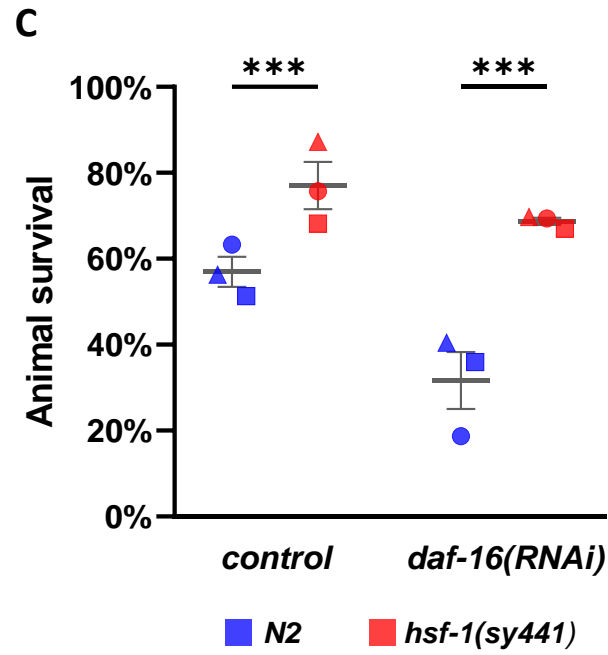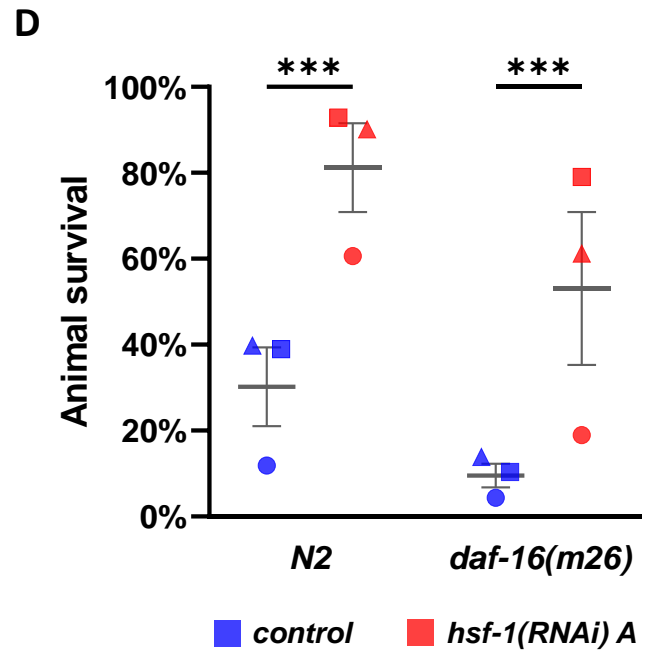

Supplement: Supplementary file 3 — Figure S3. [file ACEL-23-e14246-s015.pdf]

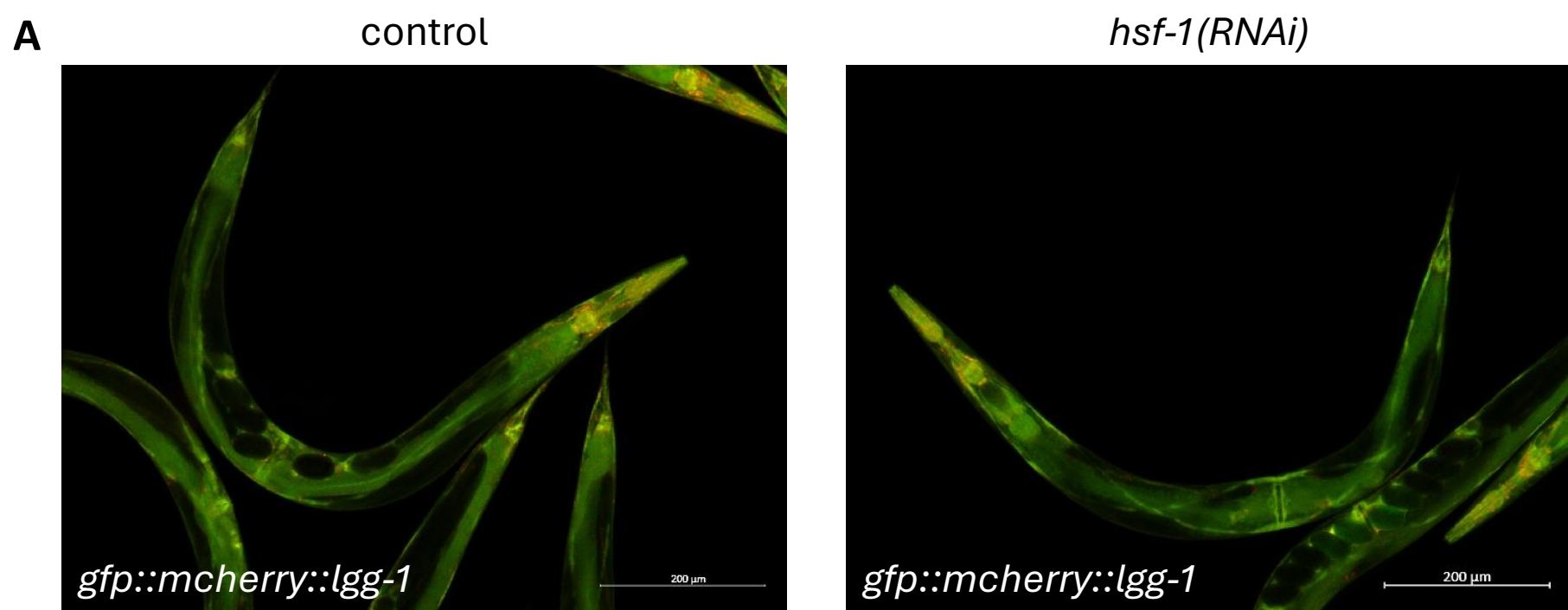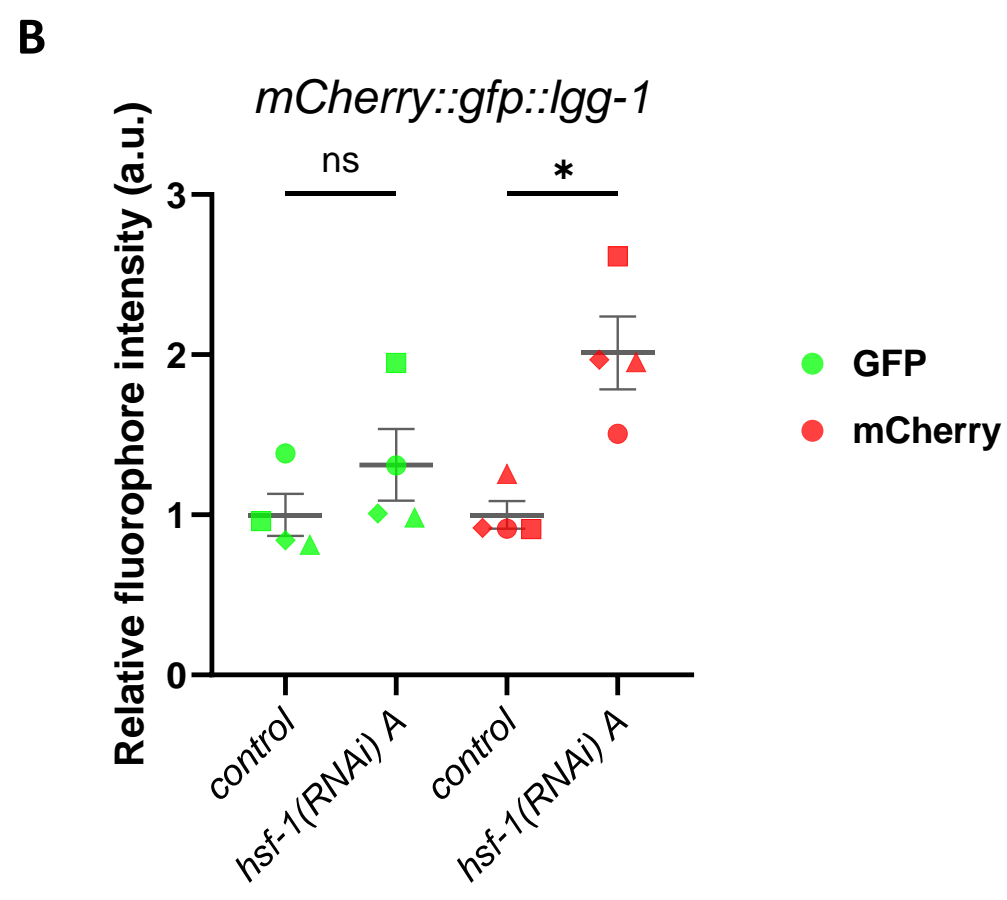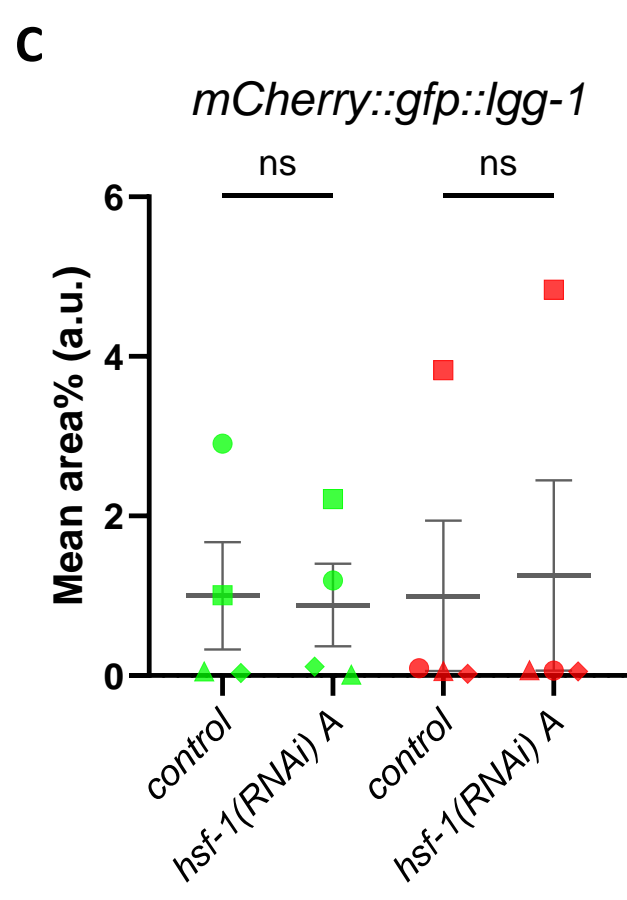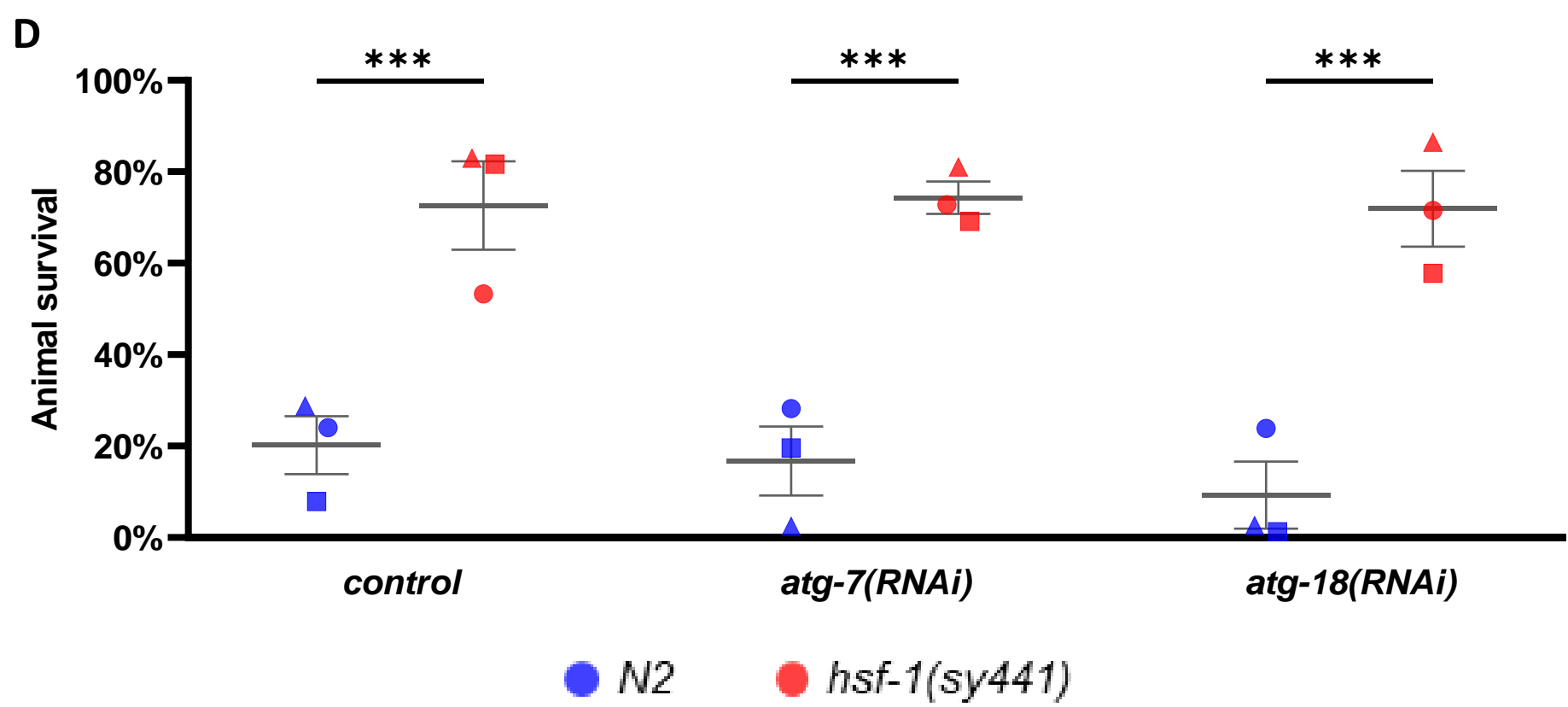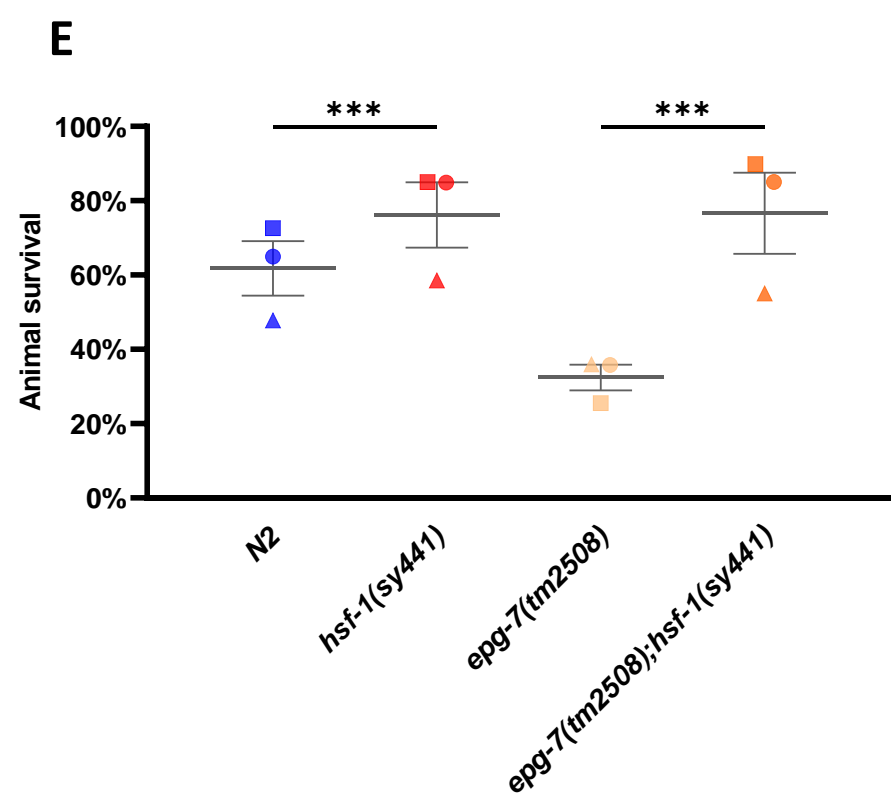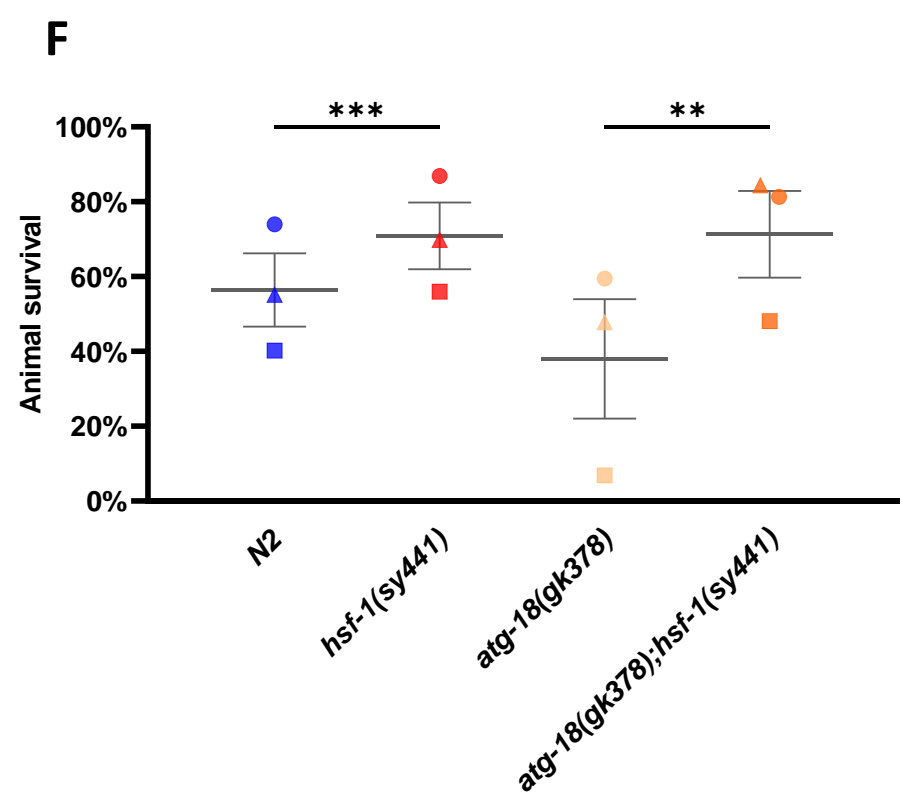

Supplement: Supplementary file 4 — Figure S4. [file ACEL-23-e14246-s012.pdf]

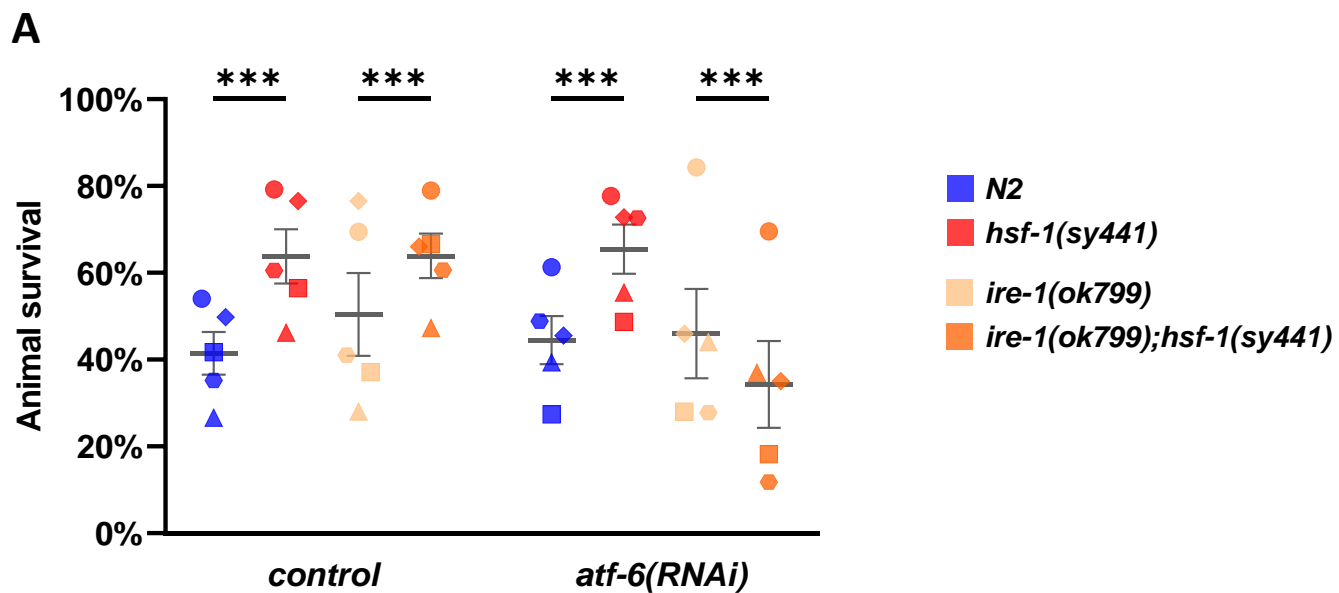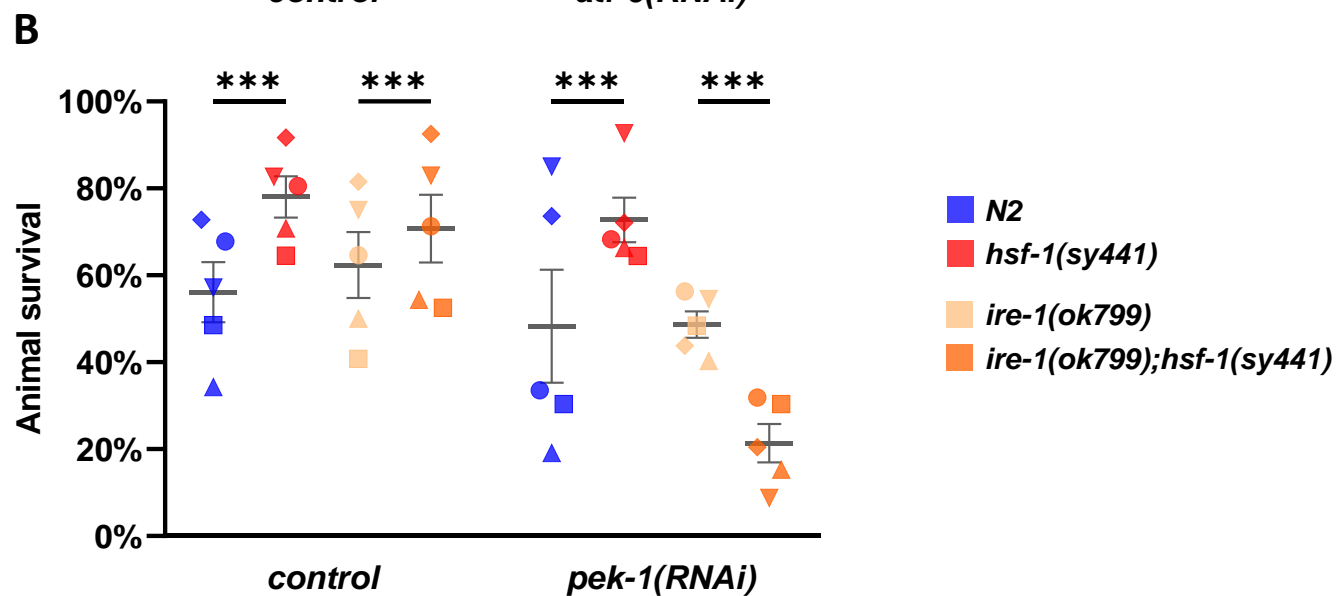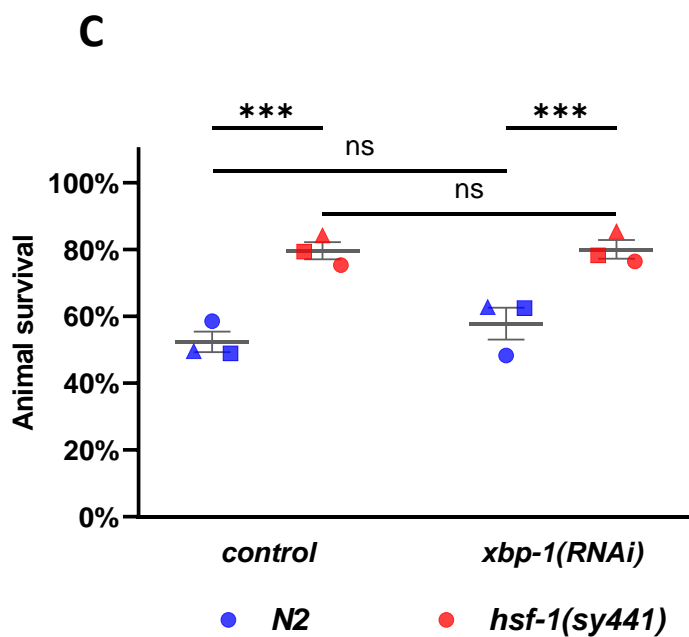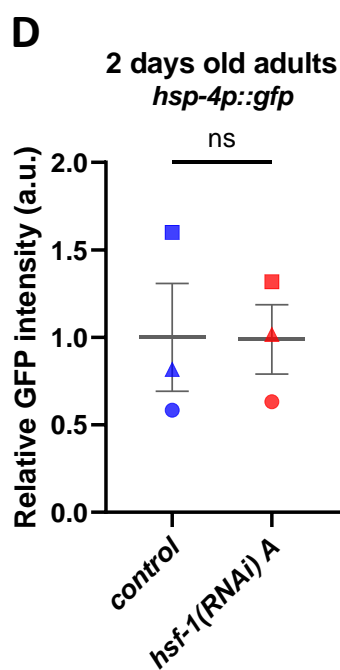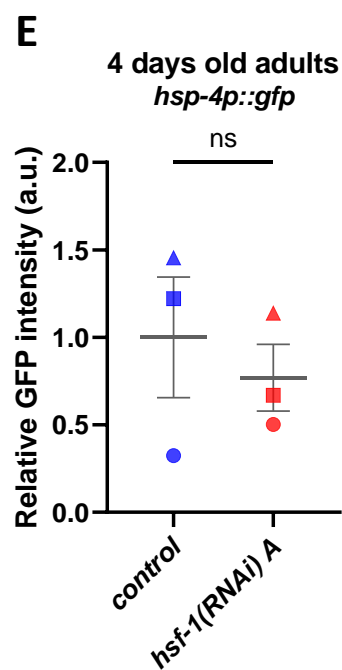

Supplement: Supplementary file 5 — Figure S5. [file ACEL-23-e14246-s016.pdf]
