# Supplementary material for: Age‐dependent heat shock hormesis to HSF‐1 deficiency suggests a compensatory mechanism mediated by the unfolded protein response and innate immunity in young Caenorhabditis elegans
Source: Aging Cell. 2024 Jun 19;23(10):e14246. doi: 10.1111/acel.14246 (PMC11464127; doi:10.1111/acel.14246)
Supplement: Supplementary file 23 — Table S18. [file ACEL-23-e14246-s005.docx]

**Quantitative real-time PCR**

| **Gene name** | **Forward primer** | **Reverse primer** |
| --- | --- | --- |
| *cdc-42* | 5’-CTTCGACAATTACGCCGTCAC-3’ | 5’-CGAAATTTCAGGCACCCATTTTTC-3’ |
| *hsp-70a* | 5’-CGGTATTTATCAAAATGGAAAGGTT-3’ | 5’-TACGAGCGGCTTGATCTTTT-3’ |
| *hsp-70b* | 5’-TGCACCAATCTGGACAATCT-3’ | 5’-TCCAGCAGTTCCAGGATTTC-3’ |
| *hsp-16.2* | 5’-TCCATCTGAGTCTTCTGAGATTGTTA-3’ | 5’-TCTTTCTTTGGCGCTTCAAT-3’ |
| *hsp-16.11* | 5’-GGCTCAGATGGAACGTCAA-3’ | 5’-GCTTGAACTGCGAGACATTG-3’ |
| *lips-11* | 5’-GGTCAACCGGAATTGCTGAAA-3’ | 5’-ATATTGCTGTGCACTGAGGC-3’ |
| *asp-8* | 5’-GGACTAGTTGCCCTCTGCTC-3’ | 5’-CTGTCCGTAGCGTCCTTCTC-3’ |
| *osg-1* | 5’-AAACGTGTCGACAGTACCCC-3’ | 5’-CACCGCTGCATTACCGATTG-3’ |
| *pals-6* | 5’-GTTGGTCAGGCACAGGAGAA-3’ | 5’-AGCCGATGCATCACTTCCAT-3’ |
| *skr-5* | 5’-AGCAGCGAAGAGCAAGATGT-3’ | 5’-AATGGAATTGGTTCGCGCTG-3’ |
| *warf-1* | 5’-AATGCAAGATCACCAGCCGA-3’ | 5’-CCTTGTCCAGAGTGAGCGTT-3’ |
| *sod-3* | 5’-CACTGCTTCAAAGCTTGTTC-3’ | 5’-ATGGGAGATCTGGGAGAGT-3’ |
| *calu-1* | 5’-TTTCAAGGGAAAGGAACATGAC-3’ | 5’-CTTGGCAAGCTTCTCCTTTG-3’ |
| *lys-7* | 5’-GTCTCCAGAGCCAGACAATCC-3’ | 5’-CCACCGCTGTACACATTCCA-3’ |

**RNA interference**

| **Gene name** | **Forward primer** | **Reverse primer** | **Restriction enzyme sites used** |
| --- | --- | --- | --- |
| *atg-7* | 5'-TATTCTAGACTCGATGAGACGCCAAAGTG-3' | 5'-TATAAGCTTCGAAATGTGACGAACTCCCC-3' | XbaI and HindIII |
| *atg-18* | 5'-ATTTCTAGATCAGAAGGATCCACGAGTGC-3' | 5’-TGGCTCATTGGTGGGAAGTC-3’ | XbaI and HindIII |
| *ire-1* | 5’-CGGAATTCGATTAGCAGGAACCGATGGC-3’ | 5’-CGGAATTCTTGCTCGACAGGTTTTGGTG-3’ | EcoRI |
| *pek-1* | 5’-ATGGATCCTCGATTCGTGCAATGGACAC-3’ | 5’-ATGGATCCATCATCTGTCGGTGCAAACG-3’ | BamHI |
| *atf-6* | 5’-TATCTGCAGTACCTCAGAGAAGAACCGGC-3’ | 5’-TATCTGCAGTTGAACCGCCAATGTCTGTG-3’ | PstI |
| *elt-2* | 5’-ATATAGATCTACGCATCTCCAATTGAACGG-3’ | 5’-ATATTCTAGAGGAACCATCATCAACCCAGC-3’ | BglII and XbaI |
| *pals-22* | 5’-CGGTTCCATGGCTAGATGTCTTCAAATGAAAATTCAGATG-3’ | 5’-CGGTATCGATAAGCTTCAAAGTGTAAAACCATTATCAATT-3’ | TEDA cloning |
| *xbp-1* | 5’-TATCTGCAGGCCACAGCCTCAGAGAATGG-3’ | 5’-TATGTCGACAACAAGACGCTGCATAACCG-3‘ | PstI and SalI |

***C. elegans* strains**

Wild-type N2 Bristol isolate

PS3551 *hsf-1(sy441)* I.

TTV450 *hsf-1(sy441)* I.

RB545 *pek-1(ok275)* X.

TTV827 *pek-1(ok275)* X*.*; *hsf-1 (sy441)* I*.*

RB925 *ire-1(ok799)* II.

TTV833 *ire-1(ok799)* II*.; hsf-1 (sy441)* I.

TJ375 *gpIs1[hsp-16.2p::gfp]*

TTV858 *gpIs1[hsp-16.2p::gfp]; hsf-1(sy441)* I*.*

SJ4005 *zcIs4[hsp-4p::gfp]*

TTV448 *hsf-1(sy441) I.; zcIs4[hsp-4p::gfp]*

MAH215 *sqls11[lgg-1 p::mCherry::gfp::lgg-1+rol-6]*

TTV673 *slqs11[lgg-1 p::mCherry::gfp::lgg-1 + rol-6]; hsf-1(sy441)* I.

CF1553 *muls84 [sod-3p::gfp; rol6(su1006)]*

TTV793 *muls84 [sod-3p::gfp; rol6(su1006)]; hsf-1(sy441)*I*.*

VC893 *atg-18 (gk378)* V.

TTV758 *atg-18 (gk378)* V*.; hsf-1(sy441)* I.

DR26 *daf-16(m26)* I.

AGD927 *uthIs270* [*rab-3p::xbp-1s* (constitutively active) + *myo-2p::tdTomato*].

TTV950 *hsf-1(sy441)*; *uthIs270* [*rab-3p::xbp-1s (constitutively active) + myo-2p::tdTomato*]*.*
